# Phytol nanoemulsions encapsulated alginate hydrogel beads for the protection and management of alcohol-induced gastric ulcer via nitric oxide synthase and NF-κB/IL-6/TGF-β modulation

**DOI:** 10.1371/journal.pone.0327368

**Published:** 2025-07-11

**Authors:** Tamer I. M. Ragab, Abdulsalam M. Kassem, Heba M. I. Abdallah, Sally A. El Awdan, Naglaa M. Ammar, Abd El-Nasser G. El Gendy, Basma Emad Aboulhoda, Sherif M. Afifi, Tuba Esatbeyoglu, Enayat A. Omara, Abdelsamed I. Elshamy

**Affiliations:** 1 Chemistry of Natural and Microbial Products Department, National Research Centre, Dokki, Cairo, Egypt; 2 Department of Pharmaceutics and Pharmaceutical Technology, Faculty of Pharmacy (Boys), Al-Azhar University, Nasr City, Cairo, Egypt; 3 Pharmacology Department, Medical Research and Clinical Studies Institute, National Research Centre, Dokki, Cairo, Egypt; 4 Therapeutic Chemistry Department, National Research Centre, Dokki, Cairo, Egypt; 5 Medicinal and Aromatic Plants Research Department, National Research Centre, Dokki, Cairo, Egypt; 6 Anatomy and Embryology Department, Faculty of Medicine, Cairo University, Cairo, Egypt; 7 Department for Life Quality Studies, Rimini Campus, University of Bologna, Rimini, Italy; 8 Department of Molecular Food Chemistry and Food Development, Institute of Food and One Health, Gottfried Wilhelm Leibniz University Hannover, Hannover, Germany; 9 Pathology Department, National Research Center, Dokki, Cairo, Egypt; 10 Department of Natural Compounds Chemistry, National Research Centre, Dokki, Giza, Egypt; National University of Medical Sciences, PAKISTAN

## Abstract

**Background:**

Innovative treatment strategies are required for stomach ulcers because of their multifactorial nature. Nanotechnology has emerged as a promising and transformative platform for the formulation and targeted delivery of therapeutic agents.

**Methods and findings:**

The gastroprotective potential of both in its free form and encapsulated in calcium alginate beads was evaluated against ethanol-induced gastric ulceration in rats. Phytol-loaded nanoemulsions were incorporated into alginate beads to achieve controlled release. Alginate beads showed a pH-dependent release pattern. The release behavior showed a higher release rate at pH 6.8 than at pH 1.2. Phytol release kinetics followed the Korsmeyer–Peppas model, indicating a release mechanism governed by diffusion and polymer relaxation. Rats were pretreated with Phytol and/or nano-Phytol at 10 or 20 mg/kg doses administered one hour before ethanol exposure. Gastric ulcer was induced by administration of EtOH (1 mL/kg, p.o.) 0.5 h after NG-nitro-L-Arginine Methyl Ester (L-NAME) or Aminoguanidine (AMG) injection. Phytol treatment led to a reduction in ulcer index and severity and improved stomach gross morphology. Also, interleukin-6 (IL-6) gastric contents were reduced, whereas transforming growth factor β (TGF-β1) was elevated, and histopathological features were ameliorated. Western blot analysis revealed that nano-Phytol exerted greater inhibitory effects on caspase-3 and Nuclear Factor kappa B (NF-κB) than unformulated Phytol. Interestingly, Phytol’s pharmacological effects on ulcers were enhanced by its nanoformulation in a dose-dependent way without exhibiting any toxicity symptoms. Confocal laser scanning microscopy (CLSM) confirmed significantly improved tissue penetration of nano-Phytol within the stomach layers compared to the Phytol. The Phytol or nano-Phytol gastroprotective effects were modified via the co-administration of L-NAME and AMG.

**Conclusions:**

The nano-Phytol formulation significantly enhanced the gastroprotective effect of Phytol against ethanol-induced gastric ulcers, primarily through modulation of the nitric oxide (NO) synthase pathway, suppression of inflammation, and upregulation of the growth factor TGF-β1.

## 1. Introduction

Complex and multifactorial inflammatory mucosal lesions characterize gastric ulcers. These lesions manifest as necrotizing patches and hemorrhagic areas affecting both the stomach’s mucosal surface and deeper tissue layers. The pathophysiology of this condition is intricate and involves an imbalance between protective factors (mucus, bicarbonate, prostaglandins, nitric oxide (NO), growth factors, and cell renewal) and aggressive agents (hydrochloric acid, long-term use of NSAIDs, smoking, oxidative stress, and excessive EtOH consumption). Additionally, anti-secretory and cytoprotective mechanisms have prevented histamine release and acid secretion in stress-induced models while decreasing gastric acid accumulation in pylorus-ligated [1].

Several medications have been used to treat peptic ulcers. Proton pump inhibitors are thought to be the most commonly prescribed drugs. However, their use is hindered by their high cost, lack of full curative impact, and development of detrimental consequences with long-term usage [2]. Thus, an emerging interest is investigating natural products as an alternative therapy. Natural remedies for stomach ulcers have been demonstrated to have both therapeutic and preventive benefits. Phytochemical constituents of these plants have ameliorated gastric ulcers via different mechanisms, such as augmentation of the prostaglandin-cyclooxygenase pathway, diminished production of reactive oxygen species (ROS), and enhanced secretion of bicarbonate and mucus in aspirin, indomethacin, and ibuprofen-induced ulcer models. Also, anti-secretive, cytoprotective mechanisms in acetic-acid-induced ulcers prevented histamine release and acid production in stress models. In addition, there is decreased gastric acid accumulation in pylorus ligation [3].

In nature, phytol (3,7,11,15-tetramethylhexadec-2-en-1-ol) is a plentiful acyclic isoprenoid compound. It is produced by practically all species of photosynthetic organisms, including plants, microbes, and algae. It is a component of the chlorophyll molecule [4]. Despite being mostly utilized as a component in fragrances, phytol’s important biological features have recently attracted interest due to potential uses in the biotechnological and pharmaceutical sectors [4]. Recent studies using phytol revealed effects that included anxiolytic [5], metabolism-modulating, anticancer [6], antioxidant [7], promoting autophagy and apoptosis, antinociceptive [7], anti-inflammatory [8], immune-modulating [5], and antimicrobial [9]. Some of the bioactivities of phytol are also attributed to PPAR- and NFκB-mediated activities.

Loading biologically active compounds into nanosystems has emerged as one of the most significant innovative approaches for drug advancement in the past few years [10–22]. Pharmaceutical medicinal products’ effectiveness, specificity, and targeting ability are increased with nanoencapsulation [23]. Nanocarriers (NCs) enhance the payload’s presence throughout the blood and intracellular absorption and shield it from premature breakdown in the biological milieu [24]. The shape and size of NCs serve a crucial role in determining their capacity to penetrate physiological barriers and be absorbed by cells [25]. The *in vivo* performance of NCs is determined by their size and chemical makeup on the surface [26]. Since drug delivery is a recommended use, drug release mechanisms from the drug-NC formulation are equally relevant. According to the kind of NCs and the nature of the therapeutic material, the releasing mechanisms might be changed [27].

The therapeutic functions, biodegradable properties, and compatibility of naturally derived polymers make them superior to replace synthetic ones. Because they can absorb much water without losing their molecular structure, hydrogel-based natural polymers, including alginate, collagen, and gelatin, can be employed for delivering hydrophilic medicines [28]. Alginate is a promising polymer for medication and cell system delivery because of its chelating, biodegradable, immunological, and mucous adhesive features [29].

Alginate beads serve as a mucoadhesive drug delivery approach [30]. Increasing the residence period of the preparation in the stomach may be beneficial for enhancing medication absorption. Previous studies have shown the feasibility and effectiveness of using alginate beads to manage peptic ulcers. Kashid *et al*. used the polyelectrolyte complexation and ionotropic gelation process to create rebamipide alginate beads. The *in vivo* investigation demonstrated that the synthesized nanoformulation was more effective in managing ulcers than pure rebamipide [31]. Singh *et al*. used the ionotropic gelation approach to create alginate beads containing Lactobacillus acidophilus, intended to treat stomach ulcers generated by cold restraint stress. Releasing probiotics from alginate beads close to the stomach mucosa over extended periods could enable them to stick to the gastric mucosa. This allows probiotics sufficient time and space to establish colonies, resulting in a notable anti-ulcerative impact compared to free biotic therapy [32].

Researchers are still looking for safer, more effective alternatives to present medications that have fewer or no side effects, given the lack of 100% prevention of stomach ulcers, the possibility of recurrence, and the development of drug interactions in some individuals. Additionally, understanding the gastroprotective mechanisms that lead to mucus layer protection, stomach epithelium, gastric blood flow, and mucosal repair capacity is crucial for creating these novel medications. Therefore, this study was designed to optimize the benefits of consuming phytol, further explore novel pharmacological properties of this natural chemical, and fulfill the following objectives: (i) the development and characterization of the phytol nanoemulsions embedded in alginate HGBs (hydrogel beads); (ii) investigating and comparing the anti-ulcer potential of phytol and its nanoemulsion-encapsulated alginate HGBs (nano-phytol) against the EtOH-induced gastric ulcer in rats; (iii) studying the NOS (Nitric Oxide Synthase) activation and IL-6/TGF-β modulatory mechanisms *via* combination with selective (AMG) and non-selective (L-NAME) NOS inhibitors; and (iv) studying the penetration depths of the phytol and nano-phytol as compared with the reference drug, Famotidine, in the *in vivo* stomach.

## 2. Materials and methods

### 2.1. Materials

Phytol, oleic acid, sucrose, selective (amioguanidine), and non-selective (NG-nitro-L-arginine methyl ester (L-NAME)) inhibitors were supplied by Sigma-Aldrich, Inc. (St. Louis, MO, USA). Ethanol (96.0% purity) was obtained from Merck Millipore in Burlington, Massachusetts, USA. The positive control medicine, Famotidine, was procured from South Egypt Drug Industries Company (SEDICO Pharmaceutical, Cairo, Egypt). Tween 80 and Span 80 were bought from Loba Chemie (Mumbai, Maharashtra, India). CaCl_2_ was acquired from Merck Company (Rahway, New Jersey, USA). Sodium alginate (SA) was acquired from KIMIKA Co. (Tokyo, Japan). Ultrapure water with a specific resistivity of 18.2 MΩ-cm at 25 °C was produced using the Milli-Q purification system provided by EMD Millipore, Billerica (MA, USA), and utilized throughout all experimental procedures. All of the supplementary chemicals were extremely high-quality and pure analytically**—**no additional purification process needed to be performed for any of the chemicals.

### 2.2. Preparation of Phytol@NE

The phase inversion technique, a low-energy technology, was used to develop a nanoemulsion loaded with phytol (phytol@NE) at room temperature, as described in a previously published study, with minor changes [33]. Oleic acid loaded with phytol (100 mg/mL) comprised the oil (dispersed) phase, while a combination of Tween 80 and Span 80 in a ratio of 9:1 (w/w) served as the surfactant. The HLB (Hydrophilic-Lipophilic Balance) values of Span 80 and Tween 80, which stand for lipophilic and hydrophilic surfactants, respectively, were 4.3 and 15. A 3 g sucrose solution (dispersion phase) with a concentration of 25 wt% was added drop by drop into a combination of 1 g oil containing phytol and 1 g surfactant mixture while stirring magnetically using Wisestir MSH-2OD (Miliot Science, Porvoo, Finland) at a rate of 1000 rpm for one hour at room temperature.

### 2.3. Preparation of Phytol@NE-HGBs

Sodium alginate-HGBs (SA-HGBs) filled with phytol-loaded nanoemulsions were made based on SA gelatinization by the ionic gelation process reported previously [34] with minor modifications. A 2% (w/w) concentration of SA was introduced into deionized water and stirred at a speed of 400 rpm for 24 h, forming an SA reserve solution. The NE samples created in section 2.2 were combined with the SA reserve solution in a 1:1 (v/v) ratio and stirred at room temperature for 5 min to develop the nanoemulsion-filled SA solution. The solution was introduced into the CaCl_2_ solution (2.5%, w/v) using an injection syringe with a needle of 0.5 mm in diameter. The syringe pump was adjusted to a 2 mL/min flow rate, regulated by a pushing pump. The distance between the syringe needle and the CaCl_2_ solution was 15 cm. The prepared nanoemulsion-HGBs (phytol@NE-HGBs) samples were subjected to crosslinking in a CaCl_2_ solution at ambient temperature for 15 min. Following crosslinking, the phytol@NE-HGBs were rinsed twice with deionized water to eliminate impurities. Finally, the prepared samples were filtered and collected for further examination. For long-term stability, the wet phytol@NE-HGBs were subjected to freeze-drying under a vacuum using a Cryodos-50 lyophilizer (Telstar Cryodos, Terrassa, Spain) at a temperature of −50 °C for 48 h at a pressure of 0.2 mbar. The vials were sealed with rubber closures and kept at room temperature for further evaluation.

### 2.4. Characterization of Phytol@NE and Phytol@NE-HGBs

#### 2.4.1. Particle size, PDI, and zeta potential of NE and Phytol@NE.

The freshly prepared samples’ size distribution and zeta potential (NE and phytol@NE) were analyzed using a Malvern® Zetasizer Nano Zs 90 (Malvern® Instruments Limited, Worcestershire, UK). Particle sizes were determined using dynamic light scattering (DLS) at a temperature of 25 °C and a scattering angle of 90°. The ζ-potential was determined by calculating the mean electrophoretic mobility of samples diluted in Milli-Q water. The data are shown as the average ± standard deviation (SD) from three independent tests.

#### 2.4.2. Assessment of stability.

The stability of NE and phytol@NE in a colloidal suspension was monitored while stored at 25 °C. At specific time intervals, the particle size, PDI, ζ-potential, and drug content were measured for one month.

The phytol EE (entrapment efficiency) in the nanoemulsion was quantified by centrifuging 2.0 mL of NE at 15,000 rpm using a Biofuge Primo centrifuge (Sorvall, Hanau, Germany) for 20 min at 4 °C. The supernatant (containing the free phytol) was dissolved in 4 mL of ethanol and agitated for 20 min at 50 rpm and 37 °C, followed by filtration through a 0.2 μm PTFE filter and measured at a wavelength of 250 nm using a UV spectrometer (Shimadzu UV-1800, Kyoto, Japan) [35]. A calibration curve was constructed using standard amounts of phytol (25–650 µg/mL, r^2^ = 0.9989, y = 0.03019x + 0.00157) dissolved in absolute EtOH. Also, the study examined the stability of nanoemulsion at various pH levels (pH = 3–11) by evaluating the particle size.

#### 2.4.3. Transmission electron microscopy (TEM).

Phytol@NE’s particle size and shape were assessed using 80 kV TEM (JEM-1010; JEOL, Tokyo, Japan) at The Regional Center for Mycology and Biotechnology (RCMB), Al-Azhar University. Prior to taking pictures, 400 μL of the prepared nanoemulsion was applied on 400 mesh carbon-coated copper grids (CCG) and dried at room temperature [21].

#### 2.4.4. Particle diameter of Phytol@NE-HGBs.

The HGBs’ particle diameters were measured using a digital vernier caliper at a minimum distance of 0.01 mm. The results are presented as an average ± SD (n = 30).

#### 2.4.5. Field emission scanning electron microscopy (FE-SEM).

The morphology of the freeze-dried phytol@NE-HGBs was examined by an FE-SEM apparatus (Bruker Nano GmbH, a company of German origin, type vertex 5600LV SEM). Before assessing at different magnification levels, the samples were coated under vacuum by cathodic sputtering with copper and observed by a scanning electron microscope under an accelerating voltage of 20 kV.

### 2.5. Phytol encapsulation-based NE-HGBs.

The Phytol content of the Phytol@NE-HGB was quantified using the methods outlined in the earlier published study [36]. The dried phytol@NE-HGBs (20 mg) were immersed in 5 mL of citrate buffer (0.2 M, pH = 7). The mixture was agitated by an oscillating thermostatically controlled water bath shaker (100 rpm, 25 ± 2 °C, Gallenkamp, England) until all beads dissolved completely and subjected to centrifugation at 4000 rpm for 2 h at a temperature of 4 °C, then diluted 1:4 with EtOH and filtered using a 0.22 μm. The dissolved phytol was detected using a UV–visible spectrophotometer at a wavelength of 250 nm. The following equations were used to compute the entrapment efficiency (EE%) and drug loading (DL%):


$$EE\;(\mathrm{\backslash nonumber}\;\%)=\frac{Amount\;of\;encapsulated\;Phytol}{Total\;amount\;of\;Phytol\;added}$$ (1)

$$DL\;(\mathrm{\backslash nonumber}\;\%)=\frac{Amount\;of\;encapsulated\;Phytol}{Total\;wt.\;of\;nanoparticles}$$ (2)

### 2.6. *In vitro* release studies

Various mediums were used to in vitro study the phytol@NE and phytol@NE-HGBs’ pH-sensitive release using a USP apparatus II (ERWEKA, DT 600, Heusenstamm, Germany). Following the United States Pharmacopeial Convention (USP), simulated intestinal fluid (SIF, pH 6.8), simulated gastric fluid (SGF, pH 1.2) without enzyme, phosphate buffer saline (PBS), and 0.1 N HCl, respectively, were used. The freshly prepared phytol@NE-HGBs were submerged in 500 mL of release media and stirred at 37 °C at 100 rpm. At regular intervals, the aliquots were removed and replaced with a new release medium to keep the volume constant. The collected samples were centrifuged and filtered through a 0.22 μm syringe filter. The phytol content in the supernatant was measured spectrophotometrically at a λ_max_ of 250 nm. Three separate runs were used to determine the average and standard deviation.

### 2.7. *In vitro* drug release kinetics

Four widely used mathematical models—zero-order, first-order, Higuchi-order, and Korsmeyer–Peppas—were used to study the phytol release kinetics from phytol@NE-HGBs up to 60%. The following equations were used to calculate the drug release parameters using mathematical models:


$$Q_{t}=\;{\;K}_{0}.t\;Zero-order\;model$$ (3)

$$\ln(100-Q_{t})=ln100-K_{1}.t\;First-order\;model$$ (4)

$$Q_{t}=\;{K_{H}.t}^{1/2}\;Higuchi\;model$$ (5)

$$Q_{t}=\;{K_{KP}.t}^{n}\;Korsmeyer-Peppas\;model$$ (6)

Where t represents the release time in minutes, Q_t_ is the drug quantity released at time t in mg/mL, k is the kinetic constant, and *n* is the release exponent that signifies the mechanism of drug release. At *n* = 0.43, the release mechanism functions according to Fickian diffusion. When the value of *n* falls between 0.43 and 0.85, the release mechanism is determined by an anomalous transport process, which involves a combination of diffusion and polymer relaxation processes. At *n* = 0.85, the release mechanism operates under Case-II transport, exhibiting the impact of polymer relaxation on the mobility of drug molecules in the matrix. The adequacy of the fit was assessed using the correlation coefficient, R^2^ [19,37].

### 2.8. Pharmacological and biological studies

#### 2.8.1. Animals and ethical protocol.

The Animals House at the National Research Centre in Cairo, Egypt, provided male Wistar rats weighing 120–130 g. The animals were housed in polypropylene cages under standard laboratory conditions, including a 12-hour light/dark cycle, a controlled temperature of 23 ± 2 °C, and ambient humidity. They had ad libitum access to water and standard laboratory chow. The Institutional Animal Care and Use Committee of Al-Azhar University granted animal manipulation and experimentation consent, which followed the rules specified in the “Guide for the Care and Use of Laboratory Animals” (**2024−013**) and the ARRIVE guidelines. All required actions were taken to eliminate the animals’ discomfort, suffering, and weight loss during the investigation.

#### 2.8.2. Acute toxicity study.

Rats of both sexes (six rats in each group) were given phytol (oily form) and nano-phytol (pellet form) suspended in distilled water with 1% Tween 80 at graded dosages up to 5 g/kg via oral gavage. Similar amounts of 1% Tween 80 in distilled water were given to the control group. The % mortality was recorded 24 h later. Signs of behavioral changes within the first 4 h were also observed.

#### 2.8.3. Gastro-protection activity experimental design.

The rats were allocated into a total of 17 groups at random, with six animals in each group: One milliliter of the vehicle (1% Tween 80 in distilled water) was given to **Group 1** (negative control). **Group 2** (ulcer-positive control): received 96% EtOH (1 mL/kg, orally) to overnight-fasted animals [38]. **Group 3:** received the standard drug, Famotidine (20 mg/kg orally) [39]**. Group 4**: received Phytol (10 mg/kg orally). **Group 5**: received phytol (20 mg/kg orally). **Group 6**: received nano-phytol (10 mg/kg orally). **Group 7**: was treated with nano-phytol (20 mg/kg orally). **Group 8**: received L-NAME (10 mg/kg); a non-selective inhibitor of NOS [40]. **Group 9**: received L-NAME + phytol (10 mg/kg). **Group 10**: received L-NAME + phytol (20 mg/kg). **Group 11:** received L-NAME + nano-phytol (10 mg/kg), and **Group 12:** received L-NAME + nano-phytol (20 mg/kg). **Group 13**: received AMG (150 mg/kg, i.p.), a selective inhibitor of iNOS [41]. **Group 14**: received AMG + phytol (10 mg/kg). **Group 15**: received AMG + phytol (20 mg/kg). **Group 16**: received AMG + nano-phytol (10 mg/kg). **Group 17**: received AMG + nano-phytol (20 mg/kg). Gastric ulcer was induced by administration of EtOH (1 mL/kg, PO) 0.5 h after L-NAME or AMG injection. One hour after EtOH administration to all groups except the negative control group, animals were sacrificed by cervical dislocation to minimize suffering.

All required measures, including compassionate techniques for alleviating discomfort, were implemented to reduce the suffering and pain. Animals were observed daily from the onset of the investigation, with observation frequency increasing to twice daily as the study progressed. A certified laboratory animal veterinarian conducted assessments and tests. Each animal’s respiration rate ranged from weak to normal, and their mobility, posture, and behavior were also closely observed. Every day, the body weight of every animal was noted separately. Additionally, a Universal Interface Device (UID) monitor measured body temperature on average twice daily. Prior to the scheduled euthanasia and at the point of reaching humane endpoints, the animals’ body temperatures were measured using a non-contact infrared thermometer (Lasergrip 774, Etekcity Inc., Anaheim, CA, USA) while their scruffs softly held them.

At the end of the study, all rats were quickly and humanely sacrificed by cervical dislocation after undergoing anesthesia via an intraperitoneal injection of sodium pentobarbital at a dosage of 40–50 mg/kg in accordance with both the National Research Center’s (NRC) and Al-Azhar University’s safety and health committee. The animals’ stomachs were then dissected and cut open across the greater curvature.

#### 2.8.4. Ulcer number and ulcer severity.

Afterward, the stomachs were stretched out on a plastic board, rinsed with saline, and checked for mucosal lesions. Ulcer number was calculated and recorded as the number of lesions per rat. As shown in **Table 1**, gastric lesions were also rated and scored based on their severity, ranging from 1 to 5. By dividing the overall ulcer score (determined by the severity of each lesion) by the total number of lesions for each rat, the total ulcer score per rat was calculated. For every animal group, the data are presented as mean ulcer number or total ulcer score ± SEM [42]. The stomachs were then washed and conserved in 0.1M phosphate saline buffer (1:4 (w/v), pH 7.4) to produce a 20% w/v stomach homogenate, which was then stored at −80 °C until biochemical analysis [42].

**Table 1:** Evaluation and description of gastric mucosa using the ulcer scoring system.

Score	Remark
1	Petechial lesions
2	Lesions less than 1 mm
3	Lesion between 1 and 2 mm
4	Lesions between 2 and 4 mm
5	Lesions more than 4 mm

#### 2.8.5. Assessment of Interleukin-6 (IL-6) and transforming growth factor (TGF-β1).

Stomach tissue was homogenized using a homogenizer, “Medica equipment, MPW-120, Poland,” and homogenates were analyzed for the number of cytokines (IL-6, TGF-β1) using a specialized enzyme-linked immunosorbent assay (ELISA) kit (Merck, Germany) using an ELISA plate reader (Stat Fax 2200, Awareness Technologies, Palm City, Florida, USA). The manufacturer’s instructions for the ELISA rat kits were followed in these method procedures.

#### 2.8.6. Assessment of oxidative stress indicators.

Using readily accessible kits (Biodiagnostic, Giza, Egypt), the effects of the negative control, positive control, and reference drug groups, in addition to the most active groups, phytol (20 mg/kg) and nano-phytol (20 mg/kg), on the oxidative damage markers GSH (glutathione) and lipid peroxides were assessed using the methodology of Ohkawa and Ohishi and Beutler *et al.,* respectively [43,44].

#### 2.8.7. Western blotting.

Western blotting was performed for caspase-3 and NF-κB, according to Avci et al. (2017) for caspase-33 [45] & Carvalho et al. (2023) for NF-κB P65 [46]. The ReadyPrep™M protein extraction kit (total protein) provided by Bio-Rad Inc. (Catalog #163–2086) was employed according to the manufacturer’s instructions and was added to each sample of the homogenized tissues. The Bradford Protein Assay Kit (Markham, Ontario L3R 8T4, Canada) for quantitative protein analysis was provided by Bio Basic Inc. A Bradford assay was performed according to the manufacturer’s instructions to determine protein concentration in each sample [47]. 20 μg protein concentration of each sample was then loaded with an equal volume of 2x Laemmli sample buffer containing 4% SDS, 10% 2-mercaptoethanol, 20% glycerol, 0.004% bromophenol blue, and 0.125 M Tris HCl. The pH was checked and brought to 6.8. Each previous mixture was boiled at 95 °C for 5 min to ensure protein denaturation before loading on polyacrylamide gel electrophoresis.

**2.8.7.1. Protein separation by electrophoresis**

Samples were separated on a polyacrylamide gel; the procedure was abbreviated as SDS-PAGE, for Sodium Dodecyl Sulfate PolyAcrylamide Gel Electrophoresis, which is a standard technique for separating proteins according to their molecular weight. Polyacrylamide gels were performed using the TGX Stain-Free™ FastCast™ Acrylamide Kit (SDS-PAGE), provided by Bio-Rad Laboratories Inc. (Catalog # 161–0181, California, USA). The SDS-PAGE TGX Stain-Free FastCast was prepared according to the manufacturer’s instructions.

**2.8.7.2.Protein blotting**

The gel was assembled in a transfer sandwich from below to above (filter paper, PVDF membrane, gel, and filter paper). The sandwich was placed in the transfer tank with 1x transfer buffer composed of 25 mM Tris, 190 mM glycine, and 20% methanol. Then, the blot was run for 7 min at 25 V to allow protein bands to transfer from the gel to the membrane using Bio-Rad Trans-Blot Turbo.

**2.8.7.3.Blocking the membrane**

The membrane was blocked in tris-buffered saline with Tween 20 (TBST) buffer and 3% bovine serum albumin (BSA) at room temperature for 1 hr. The components of the blocking buffer were as follows: 20 mM Tris pH 7.5, 150 mM NaCl, 0.1% Tween 20, and 3% bovine serum albumin (BSA).

**2.8.7.4.Incubation with the primary antibody**

According to the manufacturer’s instructions (Santa Cruz Biotechnology, Inc.), primary antibodies of cleaved caspase-3 and phosphorylated NF-κB P65 were diluted in TBST. Incubation was done overnight in each primary antibody solution against the blotted target protein at 4 °C. The blot was rinsed 3–5 times for 5 min with TBST. Incubation was done in the HRP-conjugated secondary antibody (goat anti-rabbit IgG-HRP-1 mg goat mAb-Novus Biologicals) solution against the blotted target protein for 1 hr at room temperature. The blot was rinsed 3–5 times for 5 min with TBST.

**2.8.7.5.Imaging and data analysis quantitation**

The chemiluminescent substrate (Clarity™ Western ECL substrate, Bio-Rad; Catalog #170–5060, Bio-Rad Laboratories Inc., California, USA) was applied to the blot according to the manufacturer’s recommendation. Briefly, equal volumes were added from solution A (Clarity Western luminal/enhancer solution) and solution B (peroxidase solution). The chemiluminescent signals were captured using a CCD camera-based imager. Image analysis software was used to read the band intensity of the target proteins against the control sample beta-actin (housekeeping protein) by protein normalization on the ChemiDoc MP imager (Bio-Rad Laboratories Inc., California, USA).

#### 2.8.8. Histopathological studies.

The fundus of other stomach aliquots was carefully extracted by dissection below the gastric ridge, and the specimens were immediately fixed in a 4% formaldehyde solution for routine paraffin block preparation. After slicing through the paraffin blocks using a sledge microtome, sections measuring 5 mm were deparaffinized and stained with hematoxylin/eosin (H&E). The samples were photomicrographed using a binocular microscope (Leica, Wetzlar, Germany). A scoring system was used to score the slides under light microscopy. The system included a 0–4 scale for assessing gastrointestinal mucosal damage, leukocyte infiltration, and gastric gland distortion [48]. The healthy intact mucosa was given a score of 0; desquamated epithelium was graded as 1; desquamated superficial lamina propria was assigned a score of 2; and desquamated middle lamina propria or 2/3 reduction of gastric glands was scored as 3. Score 4 was recorded for desquamated lower lamina propria, *> *2/3 reduction of gastric glands, or exposure of submucosa. For the leukocyte infiltration, the scoring was as follows: no leukocyte infiltration was graded as 0, 2–10 per high power field (/HPF) was given a score of 1, 11–20/HPF will be scored as 2, score 3 was recorded when there were 21–30/HPF, and score 4 was given when there were *>*31/HPF. Gastric gland distortion was based on the organization criteria, epithelial vacuolation, nuclear apoptosis, and degeneration. Images were taken with an Olympus CX41 light microscope (Tokyo, Japan) and an SC100 digital camera connected to a computer system.

#### 2.8.9. Confocal laser scanning microscopy (CLSM) analysis.

*In vivo,* the study of stomach penetration depths in rats with Rhodamine B and different substances was measured by confocal laser scanning microscopy (CLSM). Rats were randomly allocated into five groups (6 rats/each group). The prepared treatments with dye were applied on the rats’ stomachs for 24h as follows: **Groups 1& 2**: received phytol 10 and 20 mg/kg, respectively. **Groups 3 & 4** received nano-phytol 10 and 20 mg/kg, respectively. **Group 5**: received the standard drug, Famotidine.

All rats were sacrificed under anesthesia, and the stomach tissues were excised and saved in a normal saline solution. Posteriorly, the tissues were fixed in paraformaldehyde for 15 min; after that, they were processed for paraffin embedding and subsequent serial sectioning, allowing the assessment of stomach penetration in different treatment groups. Rhodamine B was optically stimulated in this study using a 488 nm argon laser beam wavelength, and the fluorescence emission extending past 532 nm was analyzed (green). Imaging was then conducted using CLSM (Leica, TCS SP8, Heidelberg, Germany). Rh o-123 fluorescence intensity was analyzed in the images produced using LAS X Core 3.7.4 Software (Leica, TC SP8, Heidelberg, Germany) [49].

### 2.9.Statistical analysis

The data for the biochemical assessment, western blotting results, CLSM data, and histopathological change scoring were analyzed blindly utilizing the one-way ANOVA test using GraphPad Prism software, version 8. For ulcer score and ulcer severity, a post-hoc Kruskal-Wallis comparison test was employed. Tukey’s test was applied at *P* < 0.05 to other markers. The data is provided as mean ± SEM (n = 6).

## 3. Results

### 3.1. Characterization of phytol@NE

**Fig 1:**
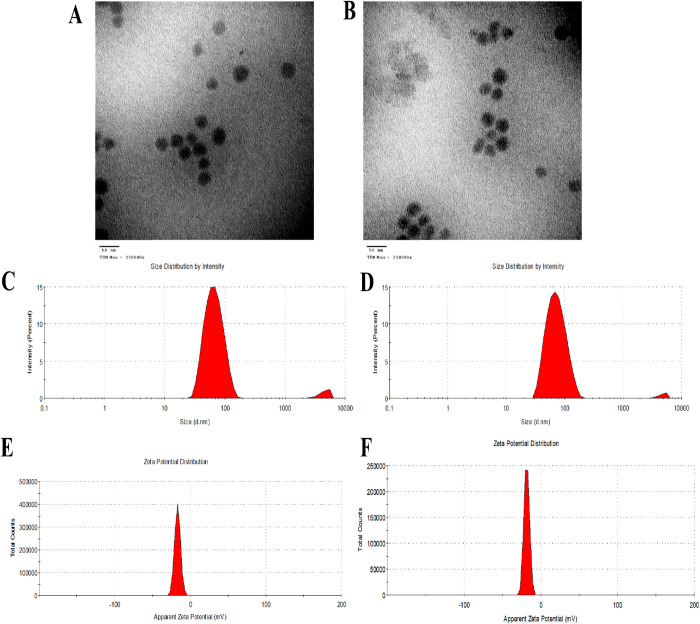
TEM, particle size, and zeta potential results. (A) free NE and (B) Phytol@NE. (C & D) The particle size of free NE and Phytol@NE. (E & F) Zeta potential distribution of free NE and Phytol@NE.

Fig 1A and B display the shape of plain NE as seen by TEM. The TEM micrographs demonstrated the nanoemulsion droplets’ distinct, uniform, and spherical morphologies. The NE and phytol@NE that were created had a diameter of around 40 nm, showing no significant difference (*P* < 0.05) between the free and the loaded NE.

The DLS examination revealed that NE particle size was 62.54 ± 5.36 nm (Fig 1C). After adding Phytol, the average diameter of Phytol@NE was slightly increased to 71.69 ± 4.68 nm, suggesting that phytol had little impact on the NE particle size (Fig 1D). At the same time, the PDI values of NE and phytol@NE were 0.12 ± 0.002 and 0.15 ± 0.040, respectively. As anticipated, the particle size measurements from DLS were greater than those seen in the TEM picture. The DLS data provides the hydrodynamic radius of particles, which includes both the core and any connected layer, depending on the intensity.

TEM relies on the particle count of a fully dried sample, with the outcomes attributed to electron scattering during sample irradiation. Zeta potential analysis was used to measure the nanoemulsion’s surface electric charge. The zeta potential values of NE and phytol@NE were −17.53 ± 0.85 mV and −15.27 ± 0.61 mV, respectively, as shown in Fig 1E and F.

**Fig 2:**
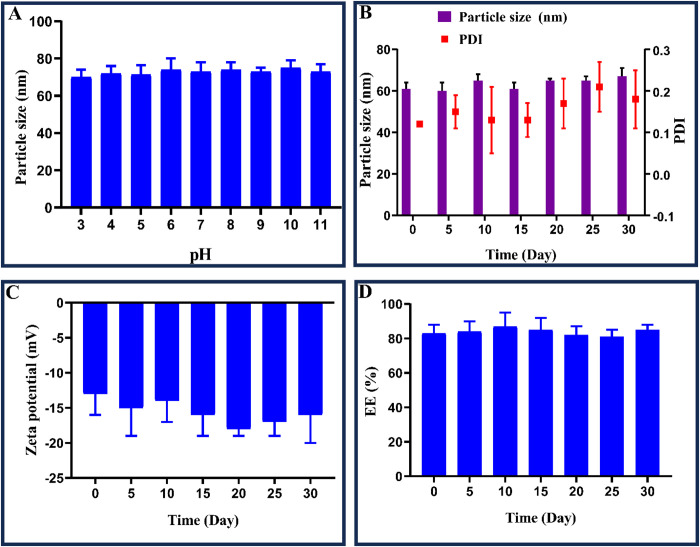
Stability over time as a function of phytol’s physicochemical parameters. (A) particle size as a function of pH, (B) particle size and PDI as a function of time, (C) ζ-potential, and (D) encapsulation efficiency (%). The mean ± standard deviation represents the data (n = 3).

Despite the decreased zeta potential, NE and phytol@NE could remain stable. Fig 2A and B indicate that the particle size remained constant with a narrow size distribution (< 0.3) over 15 days. Both NE and Phytol@NE were stable in solutions with varying pH levels, demonstrating high stability over time and under diverse pH conditions. The samples exhibited a zeta potential of around −15 mV throughout the evaluation period, as seen in Fig 2C. Moreover, no drug leakage was detected under the storage conditions with phytol EE around 80% (Fig 2D).

### 3.2. Formulation and evaluation of phytol@NE-HGBs

**Fig 3:**
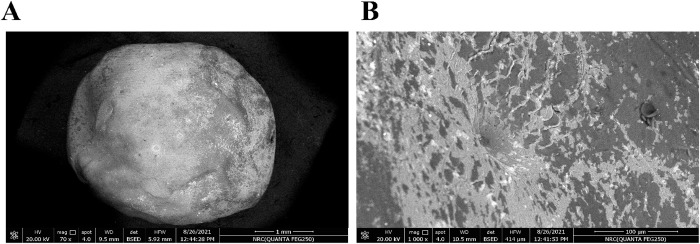
SEM of phytol@NE-HGBs. (A) Front view of a sodium alginate droplet highlighting spherical morphology and smooth surface. (B) The top perspective showed mid-detachment from the needle, focusing on the droplet’s upper hemisphere, revealing transient surface ripples caused by extrusion pressure.

The Phytol-encapsulated alginate beads exhibited particle sizes of approximately 3.87 ± 0.1 mm. Figs 3(A and B) and S1(A and B) show a standard SEM of the dehydrated bead. The HGBs were round and had a smooth surface. Freeze drying may decrease surface roughness and remove cracks. The dried beads’ average diameter was about 4 mm, displaying a relatively consistent size distribution. Size measurements taken using FE-SEM were in agreement with the data obtained using a digital vernier caliper. The possibility of entrapping functional compounds using HGBs and emulsions has been discovered. Emulsion-filled SA-HGBs were used as delivery vehicles to address the challenge of phytol’s low solubility in water despite its many biological actions. The limited solubility restricts its capacity to be absorbed and used for many applications. The investigation found that EE and DL of phytol in the emulsion-filled alginate HGBs generated using ionic gelation were 88.67 ± 0.85% and 5.28 ± 1.27 mg/g, respectively.

### 3.3. Release behavior of phytol

**Fig 4:**
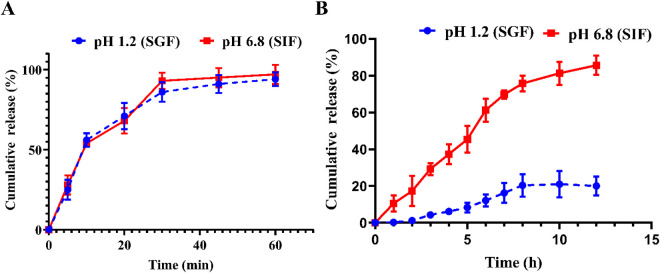
*In vitro* release profiles of phytol. (A) phytol@NE and (B) phytol@NE-HGBs in 0.1 N HCl buffer (pH 1.2, SGF) and phosphate buffer (pH 6.8, SIF) at 37 °C and 100 rpm. Phytol@NE and Phytol@NE-HGBs, the release continued for one hour and 12 hours, respectively. Data represent mean ± SD (n = 3).

The study investigated the dissolution of phytol from the phytol@NE and phytol@NE-HGBs at different pH settings (1.2 and 6.8) to evaluate the impact of medium pH on phytol release. As seen in Fig 4A, the phytol@NE exhibited over 95% release of phytol within one hour in both pH 6.8 (SIF) and 1.2 media (SGF), with no statistically significant difference (*P* > 0.05). The findings showed that the components of phytol@NE enhanced the solubility and dissolution of phytol regardless of the pH of the release medium, potentially increasing the *in vivo* bioavailability of phytol.

The suitability for delayed release of phytol under various pH levels was assessed by studying the *in vitro* release behavior in buffer solutions at 37 °C. Fig 4B demonstrates that the cumulative release of phytol ISF (pH 6.8) exceeded that in SGF (pH 1.2), suggesting that external conditions influenced the release rate of phytol. Phytol was quickly released from HGBs under neutral conditions, with a slower release rate in acidic environments. Approximately 50% of phytol was released from the emulsion-filled alginate HGBs during the first 8 h at a pH of 6.8. Equilibrium was achieved after about 12 h, releasing almost 87% of phytol from the HGBs. Meanwhile, 20% phytol was released when the pH reached 1.2 at the end of the scheduled time. HGBs may have increased corrosion in neutral environments but remain stable in acidic settings.

### 3.4. Mechanism of phytol release from phytol@NE-HGBs

The kinetics of phytol release from alginate beads at pH 1.2 and 6.8 were analyzed using Equations (3)–(6). The adequacy of the fit was assessed based on the R² values found in Table 2. The Phytol release kinetics from the alginate beads at pH 1.2 followed the first-order model (R^2^ = 0.9974). The release of phytol from alginate beads followed a non-Fickian process based on the *n* values (0.43 < *n* < 0.85). Phytol was released due to a combination of diffusion and polymer relaxation processes. The drug release kinetics from the alginate beads at pH 6.8 conform to the Korsmeyer-Peppas model (R^2^ = 0.9969). The phytol release from the alginate beads at a pH of 6.8 showed a non-Fickian mechanism where *n* = 0.8169.

**Table 2:** Release kinetics of phytol from phytol@NE-HGBs using different kinetic models.

Mechanism	Korsmeyer-Peppas		Higuchi	First-order	Zero-order	pH
	*n*	R^2^	R^2^	R^2^	R^2^	
Non-Fickian	0.721	0.989	0.873	0.997	0.845	1.2
Non-Fickian	0.816	0.996	0.695	0.905	0.783	6.8

### 3.5. Pharmacological and biological results

#### 3.5.1. Acute toxicity results.

The findings presented here imply that phytol is safe to use in the studied conditions. After being administered up to 5 g/kg, neither phytol nor its nano-form has shown any harmful effects. No signs of acute toxicity (e.g., lethargy, respiratory distress, convulsions, diarrhea, or mortality) were observed in the treated animals at the administered doses throughout the experimental period. All animals exhibited standard behavioral patterns, including typical locomotor activity, feeding, and grooming habits, with no visible adverse effects. These findings suggested that phytol and its nano-form are safe to be utilized as medicinal agents. As a result, *in vivo* investigations in the current study employed dosages of 10 mg/kg and its doubling, 20 mg/kg.

#### 3.5.2. Gross morphology.

**Fig 5:**
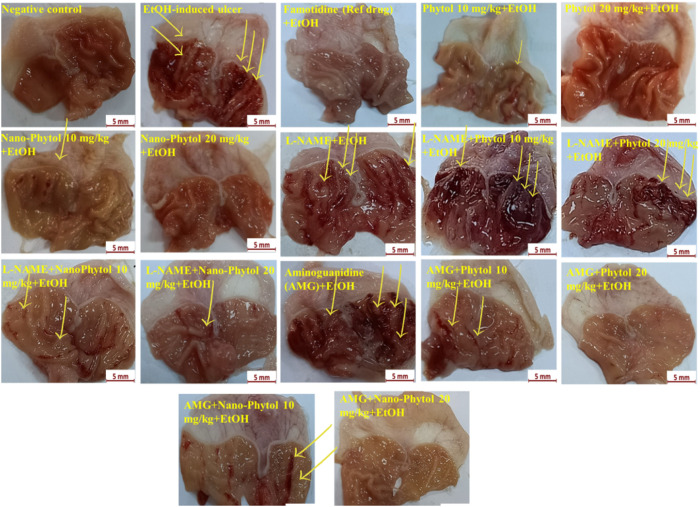
Photograph representative of macroscopical aspects of rat stomachs. Groups 3-17 were administered EtOH, as was group 2. The ulcerous regions are distinguished by arrows.

The stomachs of the positive control (ulcer) group showed signs of severe hyperemia and bleeding. The areas of gastric ulcers and lesion formation were reduced in the nano-phytol groups in a dose-dependent manner, and the Famotidine groups were compared with the control group. The administration of AMG with phytol or nano-phytol showed a synergistic improvement in stomach growth appearance. Nano-phytol (20 mg/kg) combined with AMG showed the best and nearly normal stomach appearance, comparable to the standard drug Famotidine. Administration of L-NAME deteriorated ulcers induced by EtOH. However, combining L-NAME with nano-phytol (20 mg/kg) showed a particular reduction of apparent stomach ulcers (Fig 5).

#### 3.5.3. Effect on ulcer number and ulcer severity.

**Fig 6:**
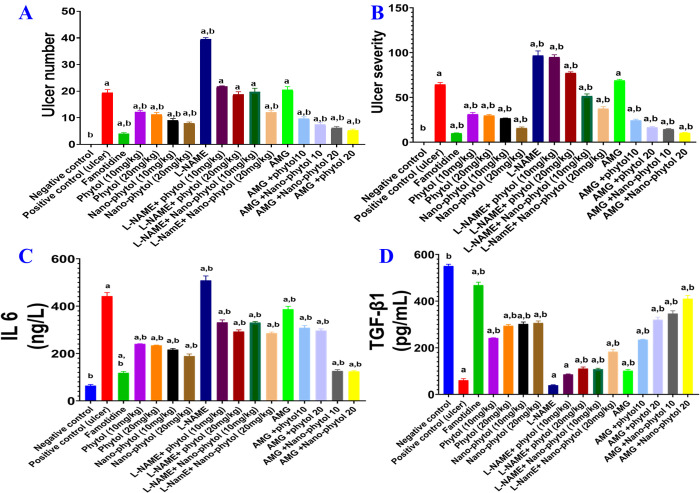
Effect of phytol and its nanoformulation on ulcer number, ulcer severity, IL-6, and TGF-β1in an EtOH-induced ulcer rat model. Results are expressed as mean ± SEM (n = 6). Statistical analysis was performed using GraphPad Prism software by applying a one-way ANOVA test and the Kruskal–Wallis comparisons test as the post-hoc test to analyze ulcer number and severity at *p* < 0.05. Tukey’s comparisons test was used as a post-hoc test to analyze IL-6 and TGF-β1 at *p* < 0.05. is significantly different from the negative control group. ^b^ is significantly different from the positive control group.

The number and severity of ulcers were recorded to further explore the ameliorative effect of Phytol and nano-Phytol on gastric ulcers induced by EtOH. Fig 6A and B depict oral administration of EtOH-induced gastric ulcer as indicated by ulcer number (Fig 6A) and severity (Fig 6B) of 19.51 ± 1.04 and 64.50 ± 2.06, respectively. However, negative control did not show any ulcers.

Compared to the EtOH group, rats given phytol and nano-phytol at doses of 10 and 20 mg/kg significantly decreased both gastric ulcer number and severity compared to the positive control group. Using nano-phytol at both doses could ameliorate the number and severity of ulcers in a dose-dependent manner (Fig 6A and B).

The AMG administration did not induce a significant change compared to the current study’s positive control group. However, treating ulcer rats with phytol or nano-phytol at both doses in combination with AMG showed synergistic improvement in ulcers in terms of number and severity. Treatment with nano-phytol (20 mg/kg) showed values of ulcer number and severity of 6.25 ± 0.48 and 10.38 ± 0.55, respectively, which were comparable to those of the standard drug, Famotidine (4.00 ± 0.39, 10.00 ± 0.41 for ulcer number and severity, respectively) (Fig 6B). The above results show that the gastroprotective effects of phytol are enhanced by nano-encapsulation in a dose-dependent manner. One mechanism underlying this gastroprotective effect could be the potentiation of the constitutive release of NO.

#### 3.5.4. Effect on the inflammatory marker (IL-6).

When comparing the positive control group to the negative control group, there was a significant (*P* < 0.05) increase in the gastric contents of inflammatory marker IL-6 by 69%. IL-6 contents were significantly (*P* < 0.05) decreased in all treatment groups except those treated with L-NAME. Treatment with nano-phytol (10 & 20 mg/kg) significantly (*P* < 0.05) decreased IL-6 levels in a dose-dependent manner. Rats treated with AMG + nano-phytol (10 & 20 mg/kg) showed the lowest levels of IL-6 comparable to Famotidine (Fig 6C). These findings explore the role of the antiinflammatory mechanism in the gastroprotective effect of phytol and its nanoformulation.

#### 3.5.5. Effect on transforming growth factor-beta1 (TGF-β1).

Fig 6D demonstrated that the gastric contents of TGF-β1 were significantly (*P* < 0.05) depressed in the EtOH-ulcer group compared to the negative control. However, treatment with phytol and nano-phytol significantly (*P* < 0.05) increased TGF-β1 level as compared to the EtOH group, with an 81% increase observed in nano-phytol (20 mg/kg). A combination of AMG with nano-phytol (20 mg/kg) showed an outstanding elevation of TGF-β1, comparable to Famotidine (85% *vs* 87%). This indicates the role that TGF-β1 plays in the phyto-induced gastroprotection, which is also related to suppression of inflammation.

#### 3.5.6. Impact on oxidative stress indicators.

**Fig 7:**
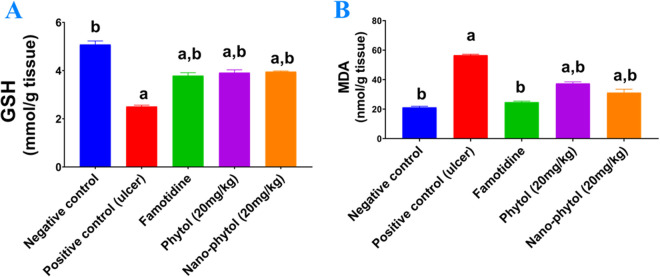
Effect of phytol and its nanoformulation on oxidative stress biomarkers. (A): reduced glutathione (GSH), (B): Lipid peroxide (MDA) in an EtOH-induced ulcer rat model. Results are expressed as mean ± SEM (n = 6). Statistical analysis was performed using GraphPad Prism software by applying a one-way ANOVA test and Tukey’s comparisons test as the post-hoc test at *P* < 0.05. ^a^ is significantly different from the negative control group. ^b^ significantly different from the positive control group.

Fig 7 shows the effect of the most pharmacologically active compounds, phytol (20 mg/kg) and nano-phytol (20 mg/kg), on markers of oxidative damage. Oral ingestion of ethanol promoted the oxidative stress state, significantly (*P* < 0.05) reduced gastric contents of glutathione (GSH) (Fig 7A), and elevated lipid peroxides (Fig 7B), reflected by MDA concentration, as compared to the negative control group. Pre-treatment of animals with phytol (20 mg/kg), nano-phytol (20 mg/kg), and Famotidine significantly (*P* < 0.05) increased GSH by 56%, 57.6%, and 51%, respectively, as compared to the positive control group. However, treatment with phytol (20 mg/kg), nano-phytol (20 mg/kg), and Famotidine showed a significant (*P* < 0.05) reduction in lipid peroxides by 34%, 45%, and 56% as compared to the positive control group. These findings indicate Phytol’s antioxidant capabilities as an additional explanation for its gastroprotective effects.

#### 3.5.7. Effect on protein expression of Caspase-3 and NF-κB in EtOH-treated rats.

**Fig 8:**
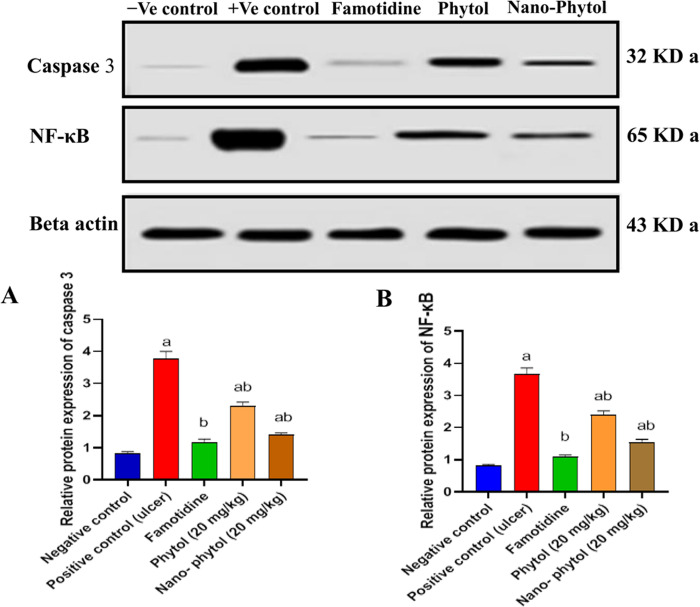
The effect of phytol and nano-phytol on protein expression in EtOH-induced ulcers in rats.

Fig 8 displays the Western blot assessment of the most significant antiulcer compounds, including phytol (20 mg/kg) and nano-phytol (20 mg/kg), on the protein expression of Caspase-3 and NF-κB in the rats induced by EtOH (the raw images were presented as supporting data as S2–S4 Figs). The results illustrated in Fig 8A display that EtOH treatment induced apoptotic injuries in gastric mucosa, as established by the significant up-regulation of the proapoptotic protein caspase-3 (*P* < 0.05), phytol, nano-phytol, and Famotidine significantly (*P* < 0.05) reduced caspase-3 by 39.36%, 62.76%, and 68.88%, respectively, relative to the EtOH group. Next, in an effort to investigate further how phytol may influence inflammation, a change in the inflammatory marker NF-kB and content in the stomach mucosa was assessed. As Fig 8(B) revealed, the inflammatory disturbances were evaluated by monitoring the protein expression of NF-κB. Ethanol intake led to a significant increase in NF-κB expression by 4.4-fold compared with the negative control group. However, phytol, nano-phytol, and Famotidine treatment attenuated the expression of NF-κB by 34.87%, 57.76%, and 70.84%, respectively, compared with the positive control group. Therefore, phytol, especially in its nanoformulation at 20 mg/kg, promoted ulcer recovery by alleviating ethanol-induced inflammation and apoptosis, highlighting its anti-inflammatory and antiapoptotic capabilities.

(A) Caspase 3 and (B) NF-κB. Each bar represents the mean ± SEM (n = 6). Significant difference from the negative group (*P* < 0.05). (b) denoted a significant difference from the positive control (ulcer) group at *P* < 0.05, employing the Tukey-Kramer multiple comparisons test after one-way ANOVA.

#### 3.5.8. Histopathological results.

**Fig 9:**
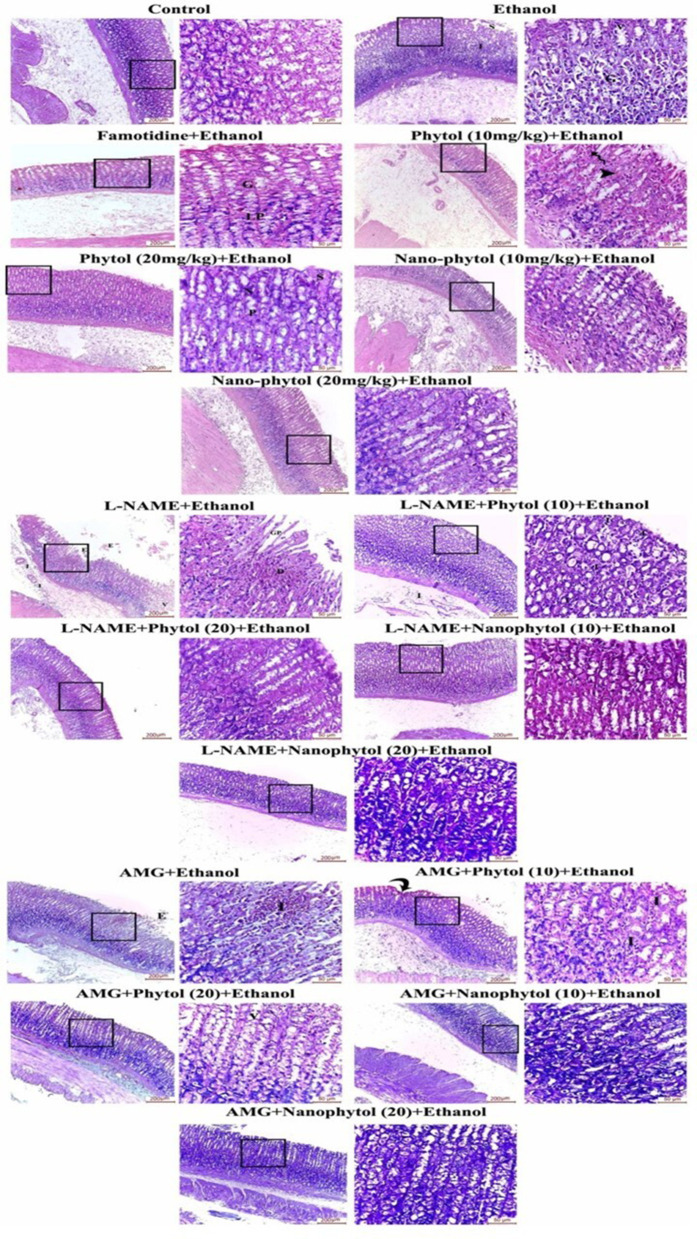
H&E-stained sections of the different study groups. (a) The control group and (b) the EtOH-treated group show erosion and sloughing of the epithelial lining (S), the base of which reveals inflammatory cellular infiltration (I). Higher magnification reveals distorted normal architecture and noticeable degeneration of the parietal cells’ gastric glands (G) and vacuolation (V). (c) The Famotidine-treated group shows regularly arranged fundic glands (G) within a preserved Lamina propria (LP). The phytol (10 mg/kg)-treated group shows few extravasated RBCs (spiral arrow) in between the glands and mild vascular congestion (arrowhead). The phytol (20 mg/kg)-treated group shows healthy fundic glands formed of preserved surface mucous cells (S) lining the gastric pits (GP) and containing acidophilic secretion, mucous neck cells (N), and parietal cells (P). The nano-phytol (10 mg/kg) and (20 mg/kg) show restoration of the gastric mucosal architecture. The fundic glands reveal an apparently normal appearance of surface mucous with acidophilic cytoplasm and oval basophilic nuclei, mucous neck cells, and parietal cells with intact rounded nuclei and eosinophilic cytoplasm. The L-NAME+EtOH group displays an area of gastric ulcer with desquamated gastric mucosal surface epithelium (E). Areas of highly vacuolated (V) gastric glands and submucosal peri-vascular infiltration (I) are also observed. Higher magnification reveals disorganized fundic glands with thinned-out, wide, empty gastric pits (GP) and areas of degenerated (D) gastric glands. L-NAME+Phytol (10)-treated group shows moderate inflammatory cellular infiltration in the submucosa (I). Higher magnification demonstrated a few clusters of inflammatory cells (I) throughout the lamina propria. The L-NAME+phytol (20)-treated group shows restoration of the gastric mucosal structure. The cytoplasm and nuclei of the surface, mucous neck, and parietal cells appear nearly normal. The L-NAME+nano-phytol (10) and (20)-treated groups show intact mucosal architecture comparable to the control groups. L-AMG + EtOH group displaying eroded surface epithelium and desquamated cells (E). Higher magnification reveals areas of degenerated gastric glands invaded by mononuclear inflammatory cells (I). The AMG+phytol (10)-treated group shows limited areas of epithelial erosion (curved arrow) and scattered inflammatory foci (I). The AMG+phytol (20)-treated group shows an improved gastric structure with only some parietal cells with vacuolated cytoplasm (v) and apoptotic nuclei. The AMG + nano-phytol (10) and (20)-treated groups show preserved parietal cells occupying the side wall of all parts of the gastric gland. In contrast, strongly basophilic chief cells are located mainly in its basal part. Minimal distortion of the gastric glands is observed. The histopathological results support the biochemical findings regarding enhancing Phytol’s gastroprotective properties by nano-encapsulation.

Histopathological assessment of H&E-stained sections revealed typical histological architecture for the gastric fundic mucosa (Fig 9). The EtOH-treated group showed erosion and sloughing of the epithelial lining, the base of which reveals inflammatory cellular infiltration. The gastric glands appeared distorted, and the parietal cells were degenerated and vacuolated. The Famotidine (standard)-treated group showed regularly arranged fundic glands within a preserved Lamina propria. The phytol (10 mg/kg) treated group revealed a few extravasated RBCs between the glands and mild vascular congestion. The phytol (20 mg/kg) treated group showed healthy fundic glands of preserved surface mucous cells lining the gastric pits and containing acidophilic secretion, intact mucous neck cells, and parietal cells. The nano-phytol (10 mg/kg) and (20 mg/kg) restored the gastric mucosal architecture. The fundic glands showed mucous neck cells, parietal cells with intact rounded nuclei, eosinophilic cytoplasm, surface mucous with acidophilic cytoplasm, and oval basophilic nuclei that appeared normal (Fig 9).

The L-NAME + EtOH group displayed areas of gastric ulcer with desquamated gastric mucosal surface epithelium, areas of highly vacuolated gastric glands, and submucosal peri-vascular infiltration. The fundic glands appeared disorganized with thinned-out, wide, empty gastric pits. The L-NAME + phytol (10)-treated group showed moderate inflammatory cellular infiltration in the submucosa and a few clusters of inflammatory cells throughout the lamina propria. The L-NAME + phytol (20)-treated group restored the gastric mucosal structure with normal surface mucous cells, mucous neck cells, and parietal cells. Meanwhile, the L-NAME + nano-phytol (10) and (20) treated groups showed intact mucosal architecture comparable to control groups (Fig 9).

The AMG + EtOH group displayed eroded surface epithelium and desquamated cells. Higher magnification reveals areas of degenerated gastric glands invaded by mononuclear inflammatory cells. The AMG + phytol (10)-treated group shows limited areas of epithelial erosion (curved arrow) and scattered inflammatory foci. The AMG + phytol (20)-treated group showed improvement in the gastric structure with only some parietal cells with vacuolated cytoplasm and apoptotic nuclei. The AMG + nano-phytol (10) and (20)-treated groups showed preserved parietal cells occupying the side wall of all parts of the gastric gland. In contrast, strongly basophilic chief cells are located mainly in its basal part. Only minimal distortion of the gastric glands was observed (Fig 9). The scoring of these histopathological changes was summarized in Table 3.

**Table 3:** Scoring of histopathological changes in the gastric tissue.

Group	Gastric mucosal injury	Leucocyte infiltration	Distorted gastric glands
Negative control	**−**	**−**	**−**
Positive control (EtOH)	**+++**	**++**	**+++**
Famotidine + EtOH	**+**	**−**	**−**
Phytol (10 mg/kg) + EtOH	**+**	**+**	**−**
Phytol (20 mg/kg) + EtOH	**+**	**−**	**−**
Nano-phytol (10 mg/kg) + EtOH	**+**	**−**	**−**
Nano-phytol (20 mg/kg) + EtOH	**−**	**−**	**−**
L-NAME + EtOH	**+++**	**++**	**+++**
AMG + EtOH	**+++**	**++**	**+++**
L-NAME + phytol (10 mg/kg) + EtOH	**+**	**+**	**++**
L-NAME + phytol (20 mg/kg) + EtOH	**+**	**−**	**+**
L-NAME + nano-phytol (10 mg/kg) + EtOH	**−**	**−**	**+**
L-NAME + nano-phytol (20 mg/kg) + EtOH	**−**	**−**	**+**
AMG + phytol (10 mg/kg) + EtOH	**+**	**++**	**+**
AMG + phytol (20 mg/kg) + EtOH	**+**	**+**	**+**
AMG + nano-phytol (10 mg/kg) + EtOH	**−**	**−**	**+**
AMG + nano-phytol (20 mg/kg) + EtOH	**−**	**−**	**+**

#### 3.5.9. CLSM visualization.

**Fig 10:**
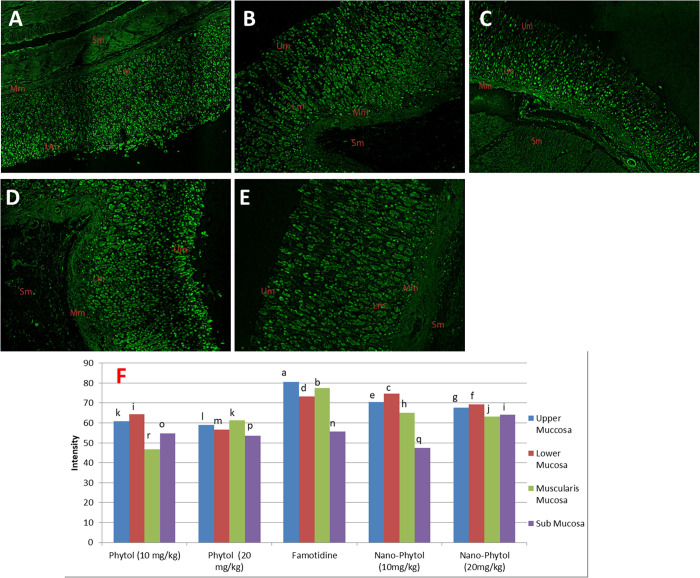
Confocal laser scanning microscopy (CLSM) image of rat stomach tissue treated.

The penetration depths of the two dosages of phytol (10 and 20 mg/kg), nano-phytol (10 and 20 mg/kg), and the reference drug (20 mg/kg), were shown based on the comparative CLSM investigation results (Fig 10). Nano-phytol (10 and 20 mg/kg) was highly permeated through the four layers of the stomach: 1^st^ (the upper mucosa), 2^nd^ (the lower mucosa), 3^rd^ (the muscularis mucosa), and 4^th^ (the submucosa), in which its penetration decreased in the sequence of 1^st^ > 2^nd^ > 3^rd^ > 4^th^ and this is deduced by reducing the darkness of the rhodamine (Fig 10A and B). Otherwise, the phytol at the two doses permeated through the layers of the stomach, but its penetration depth was lower than that of the nano-phytol within the two doses (Fig 10C and D). Famotidine is excreted as a standard drug that significantly permeates through the stomach layers, as deduced via rhodamine darkening (Fig 10E).

The comparative quantitative estimation of fluorescent intensity was performed and represented in Fig 10F. These fluorescent intensity data revealed that phytol and nano-phytol at their two doses (10 and 20 mg/kg) have good reach to all four stomach layers, as evidenced by the intensity of each layer in the confocal image. Phytol at the lower dose (10 mg/kg) exhibited significant intensities in the four layers of the stomach with values of 60.87, 64.34, 46.7, and 54.75, but lower than Famotidine, which showed intensities of 80.7, 73.3, 77.46, and 55.7, respectively. Also, phytol at the higher dosage (20 mg/kg) showed good penetration throughout the four layers within the respective values of 59.0, 56.6, 61.2, and 53.6. These results revealed that the penetration of phytol is almost the same within the two dosages and lower than that of Famotidine.

On the other side, the nano-phytol at the lower dosage (10 mg/kg) exerted stronger penetration than that of phytol, at its two dose levels in the four stomach layers within the respective intensity values of 70.5, 74.5, 65.2, and 47.53. Furthermore, the nano-phytol within the high dose (20 mg/kg) showed a similar penetration pattern at the intensity of 67.6, 69.2, 63.2, and 64.1, and it was also higher than the phytol penetration at both doses. The findings showed that nano-Phytol showed stronger penetration than phytol but less than Famotidine.

(A) Rhodamine-loaded; (B) nano-phytol 10 mg/kg; (C) nano-phytol 20 mg/kg; (D) phytol 10 mg/kg; (E) phytol 20 mg/kg, and (E) Famotidine showing the depth of penetration. Fluorescent intensity of tested substances in layers of the stomach. Similar letters are not considered significantly different from each other, while different letters are considered significantly different. Upper mucosa (Up), lower mucosa (Lm), muscularis mucosae (Mm), submucosa (Sm).

## 4 Discussion

Nanotechnology could overcome Phytol’s limitations, which restrict its functional practicality, such as a lack of long-term stability, high volatility, and poor aqueous solubility. Phytol exhibits high solubility in hydrophobic environments, such as the oil phase. Thus, oil-in-water nanoemulsions (O/W) can potentially serve as a viable nanocarrier for Phytol [50]. Phytol-loaded nanoemulsions were developed using the phase inversion technique. Tiny droplets develop as the system goes through an inversion point during dilution because the interfacial tension at the oil-water contact is significantly reduced. For the purpose of causing the subsequent production of spherical beads, sucrose is used as a small molecule thickener to raise the viscosity of the NE slightly. Viscosifying the dispersion phase has been shown to reduce the size of the nanodroplets, which improves the stability of the NE over the long term by diminishing droplet coalescence and gravitational separation [33]. The Phytol@NE exhibited good stability for a long duration, which may be attributed to the viscosity of the dispersion medium and stabilizing surfactants that keep the droplets from aggregation.

By using encapsulation, users can prevent direct access to specific object components, preventing them from accessing the state values of all the variables in that object. Sodium alginate is a frequently used polymer for encapsulation due to its ability to form a highly customizable, nontoxic, and biocompatible matrix for essential oil preservation. The developed alginate beads containing Phytol-loaded nanoemulsions were created by gelatinizing sodium alginate using an ionic gelation technique. SA has a protective effect by coating the surface of the gastrointestinal tract. Thus, it might be beneficial in treating stomach and esophageal ulcers and bleeding [51]. Alginate has been suggested as an effective, non-erosive therapy for reflux disease and may help treat upper gastrointestinal disorders [52]. Also, alginate was reported to be poorly absorbed via the gastrointestinal system and eliminated in the feces; it may benefit both the upper and lower gastrointestinal tracts [52]. Moreover, alginate has shown efficacy in treating experimental colitis and radiation-induced colon damage [53].

The effect of alginate on alcohol-induced gastric ulcers in rats was investigated in order to test our hypothesis that it could alleviate gastric ulcers. The Phytol@NE-HGBs exhibited a high loading and encapsulation efficiency, suggesting that most of the dissolved Phytol was successfully loaded. The findings were consistent with prior research and demonstrated the suitability of emulsion-filled HGBs for encapsulating hydrophobic active compounds [54]. Consequently, the results showed that the SA-HGBs filled with emulsion had a high encapsulation efficiency and showed tremendous potential as Phytol NCs. The contraction of the alginate molecular chain under acidic conditions resulted in a denser hydrogel shell and a more effective prolongation of release.

Meanwhile, the degree of dissociation of the SA–COOH groups increased with increasing pH, resulting in stronger repulsion between the single –COO groups. These findings are explained by the fact that under acidic conditions, the –COO of alginate transformed into –COOH, and the ionization degree declined [55]. This action encouraged the alginate beads to expand and the porosity to rise. Other investigations have verified this trend [56,57]. According to their findings, a higher pH caused the release from SA beads to occur more quickly. Consequently, the beads swelled after the basic pH trials. Thus, Phytol may be effectively conserved in NE-HGBs and released in physiological environments. Diffusion and polymer relaxation processes collaborated to cause the release of Phytol, as shown by the Korsmeyer-Peppas model’s release exponent, which was more than 0.43 and less than 0.85.

The effectiveness of orally administered medications may be improved by prolonging the residence time in the stomach and adjusting how the drug is released based on the therapeutic need [30]. The low aqueous solubility of Phytol reduces the time available for drug dissolution, affecting drug absorption. To address this issue, the formulation of nanoemulsion embedded in alginate beads was employed to increase Phytol’s absorption. This was achieved by prolonging the residence time of the drug [58].

The mucoadhesive delivery technique improves medication absorption at a particular site of action by sticking to the mucosal layers in that area [31]. As a drug delivery system, the alginate beads utilize mucoadhesive alginate polymer that adheres to the epithelial lining of the stomach wall, resulting in an improved stomach residence time [58]. This could improve Phytol’s effectiveness by limiting systemic adverse effects and increasing localized action on the ulcer.

The persistent release of the Phytol from the produced nanosystem was caused by forming a 3-D gel network resulting from the interaction between calcium ions and alginate. This gel network prevented the diffusion of drug molecules, leading to the sustained release of the drug. The antiulcer efficacy of the drug delivery system for stomach ulceration could be improved by the gradual release of the Phytol from the drug delivery carrier [59]. It aids in sustaining a therapeutic level of the medication in the stomach for an extended duration, facilitating sustained therapeutic actions that promote the healing of ulcers [60]. Overall, releasing the drug slowly with mucoadhesive properties could provide continuous protection and support the healing process of the ulcerated tissue.

This study investigated Phytol and its nanoformulation for their protective activity against gastric ulcers induced in rats via acute oral administration of EtOH. Pretreatment with Phytol and its nanoformulation reduced EtOH-induced stomach lesions dose-dependently. The effects of Phytol and nano-Phytol on stomach healing were diminished upon administration of the non-selective NOS inhibitor, L-NAME. However, the selective iNOS inhibitor, AMG, boosted Phytol’s effect on ulcer healing.

Initially, a toxicological study was conducted to establish a safe treatment range for using *in vivo* techniques to avoid side effects and/or unfavorable consequences [61]. Neither Phytol nor nano-Phytol showed signs of mortality or behavioral changes, and they were found to be nontoxic up to doses of 5 g/kg body weight. The Organization for Economic Cooperation and Development classified the median lethal dosage (LD_50_) as greater than 2 g/kg based on guideline No. 425 for acute oral toxicity tests [62]. All dosages below this value should be considered safe agents for the compound. Thus, 10 and 20 mg/kg were the two dose levels chosen in the current investigation.

EtOH-induced gastric ulcers are a well-established model that has long been used to screen gastroprotective agents. It has the advantage over other gastric ulcer models in that it resembles the pathological features of an acute human peptic ulcer state. EtOH also initiates microvascular damage by decreasing blood flow, producing more pro-inflammatory cytokines and ROS, and reducing cellular antioxidant levels [63]. Due to its necrotizing properties, EtOH degrades the epithelial layers on the surface, exposes the mucosa to the action of the proteolytic and hydrolytic effects of gastric HCl, and prevents mucosal prostaglandin release. It takes an hour after alcohol consumption to notice stomach lesions. Alcohol-induced lesions occur as blackish lesions clustered in patches of varied sizes that are usually parallel to the main axis of the stomach. The severity of mucosal inflammation has often been measured by counting the number of hemorrhagic spots or by grading the mucosal lesions [64]. In parallel, the current findings showed that EtOH administration successfully induced acute gastric ulcers and multiple lesions. These lesions were apparent macroscopically and were reflected by increased ulcer number and severity.

In contrast, pretreatment of ulcerogenic rats with Phytol demonstrated a dose-dependent decrease in ulcer number and severity and improved the macroscopical aspects of ulcer lesions and inflammation. Remarkably, Phytol as a nanoform showed better protective actions at a higher dose (20 mg/kg) than the standard drug Famotidine. Phytol is one of the constituents of some plants, such as *Biebersteinia multifida*. The gastroprotective effect of this plant was previously demonstrated against EtOH-induced ulcers and was attributed to its antioxidant effect [65]. Also, *Lantana camara’s* methanolic extract effectively healed gastric and duodenal ulcers in rats [66]. However, to our knowledge, Phytol as a pure phytochemical has not been investigated previously for its gastro-protective activity. Hence, in this study, the antiulcer effect of Phytol in rat stomachs is explored for the first time. Moreover, the formulation of this phytochemical as a nanoform enhanced its gastroprotective activity without causing any toxic signs or behavioral changes.

The NO molecule is crucial for the process of gastric ulcer healing. Its production improves the pH of the gastric environment, which is necessary for promoting ulcer healing. Mild stimulation of the gastric mucosa can activate NO synthesis and release, promoting the gastric alkaline response and adaptive cell protection. Other underlying pathways of NO mucosal protection are modulating mucosal blood flow, repair of mucosal injury, and mucus and bicarbonate secretions [67]. On the contrary, inhibition of NO slows the healing of ulcers by decreasing the restoration of gastric blood flow at the ulcer margin [68]. NOS is the primary enzyme that synthesizes NO. Three subtypes of NOS have been identified in the gastrointestinal tract: nNOS (neuronal nitric oxide synthase), eNOS (endothelial nitric oxide synthase), and iNOS. NO, made up of nNOS or eNOS, controls the blood flow and mucous secretion and acts as a defensive barrier to the stomach mucosa, preserving its integrity. On the other hand, under oxidative stress and some cytokines, NO was produced by iNOS in huge quantities, contributing to the inflammatory response-related tissue damage and ulcer formation [69]. So, understanding both the beneficial and harmful effects of NO in the context of gastric ulcers is necessary to improve the clinical outcomes of this disease.

In the present study, in an attempt to elucidate the potential role of NO in the antiulcer effect of Phytol in gastric ulcerogenic rats, the interactions with two different types of NO modulators were investigated: L-NAME, which is a non-selective NOS inhibitor, and AMG, which is a selective inhibitor of iNOS.

Injection of L-NAME concurrently with EtOH significantly deteriorated the number and severity of gastric ulcers as compared to the positive control group. This indicated the involvement of NO inhibition in EtOH-induced ulcers. In parallel, it was previously described that L-NAME administration had increased the damage that EtOH caused to the stomach mucosa and decreased its blood supply due to deprivation of NO-induced gastric protection [70,71]. Treatment of rats with L-NAME along with Phytol or its nanoformulation abolished their gastroprotective effect in all groups except for the group of rats treated with nano-Phytol at the higher dose (20 mg/kg), which showed a noticeable decrease in ulcer number and severity as compared to the positive control group. However, this gastroprotective effect was still lower than that of the ulcer rat group, which was treated only with nano-Phytol (20 mg/kg). This indicates the detrimental impact of L-NAME and the involvement of NO in Phytol-induced gastroprotection, consistent with previous studies [59–60].

However, iNOS can create a high level of inducible NO upon activation by inflammatory cytokines, which can cause injury to the gastric mucosa and disruption of the vascular microcirculation [72]. Pretreatment of animals with AMG, the selective iNOS inhibitor, Phytol, and nano-Phytol further potentiated their gastroprotective effect in a dose-dependent manner. Interestingly, animals were given Phytol nanoformulation (20 mg/kg), and AMG showed the best protection. Thus, it could be inferred that inhibiting the harmful iNOS-derived NO while maintaining the beneficial cNOS (constitutive nitric oxide synthase)-induced one is closely associated with the protective effect of Phytol against EtOH-induced gastric injury. In parallel, previous studies have described similar actions for AMG [40,73]. This strengthens the argument that Phytol works by modulating the NOS pathway, and further research can isolate the specific pathway (for NO production) that Phytol affects.

Changes in pro-inflammatory cytokines and gastric mucosal defense factors are strongly linked to the complicated pathogenic pathways of EtOH-induced gastric ulcers. The EtOH-induced stomach ulcer is mediated by an excessively high inflammatory response, as evidenced by the increased production of pro-inflammatory cytokines such as IL-1β (interleukin-1β) and TNF-α and the reduced expression of anti-inflammatory cytokines such as IL-10 (interleukin-10) [74]. One of these inflammatory cytokines, IL-6, is considered to have a genetic pleiotropic activity connected to tissue damage and inflammation. It was also essential in regulating the severity of stomach ulcers [75]. The current results showed that the EtOH-induced ulcer group had considerably greater levels of gastric IL-6, consistent with earlier research on the pro-inflammatory action of EtOH [76,77].

On the other hand, treatment with Phytol and nano-Phytol decreased the gastric contents of IL-6 according to a dose-dependent pattern. In parallel, previous findings implied that Phytol suppressed the inflammatory response by preventing neutrophil migration, which is partly brought on by oxidative stress, decreasing IL-1β and TNF-α levels, and other factors in different inflammation rat models, e.g., carrageenan [8] and formalin-induced paw edema models [78]. Furthermore, Phytol significantly reduced inflammation in a mouse model of arthritis induced by the complete Freund’s adjuvant [78]. The combination of AMG with Phytol potentiated its anti-inflammatory effect, which is unsurprising due to the suppression of iNOS-induced NO overproduction.

There might be a direct relation between iNOS inhibition and the anti-inflammatory activity of Phytol. The excessively produced NO combines with superoxide radicals to form reactive nitrogen species (RNSs), including peroxynitrite and dinitrogen trioxide. These RNSs start specific oxidative stress and related inflammatory pathways, resulting in a complex disease. Thus, Phytol and its enhanced nanoformula also exhibited their anti-inflammatory activity via inhibition of the detrimental production of NO by iNOS [79]. In parallel with current results, inhibition of iNOS in patients with ulcerative colitis decreased mucosal TNF-α and IL-6 production and promoted ulcer healing and positive prognosis [80].

On the other hand, in rats, inhibition of the beneficial cNOS enzyme resulted in increased leukocyte-endothelial contact and inflammation. This is consistent with the current observation that L-NAME injection diminished Phytol’s anti-inflammatory effect, indicating the importance of sustaining small amounts of NO in the overall gastroprotection of this compound.

NO has a significant role in suppressing inflammation associated with gastric lesions. It regulates leukocyte adhesion to vascular endothelium and decreases myeloperoxidase activity (an indicator of neutrophil number) and tissue damage. NO also prevents the synthesis of superoxide anion and hydrogen peroxide, which are involved in further mucosal damage. NO-induced mucosal protection was also correlated to inflammatory cytokine inhibition such as IL-6, IL-1β, and NFκB [80]. Previous studies showed that a NO-releasing aspirin derivative provided gastric protection in EtOH-treated rats by showing anti-inflammatory action [81].

TGF-β1 is a key growth factor that regulates several biological processes, such as cell adhesion, proliferation, and the production of extracellular matrix proteins. It possesses a healing effect on gastric ulcers, eventually by inducing cell migration and enhancing vascular proliferation [82]. This cytokine may also promote angiogenesis by generating VEGF, which is essential for tissue regeneration and wound repair [83]. TGF-β1 promotes gastrointestinal ulcer healing by binding to its transmembrane serine/threonine kinase receptors and activating various intracellular signaling pathways. Consistent with our results, previous reports have revealed reduced TGF-β1 levels and expression in the gastric mucosa of EtOH-ulcer rats [84,85]. Pretreatment of ulcer rats with Phytol significantly increased gastric TGF-β1 levels. This indicates that TGF-β1 plays a critical role in the phyto-induced gastroprotection.

It was also clear that TGF-β1 and inflammatory pathways are closely related to accomplishing healing from stomach ulcers. It was shown that the mucosal TGF-β1 level was lower in patients with *H. pylori*-associated gastric ulcers, and this drop in TGF-β1 levels was linked to a greater level of inflammation. This led to an increase in oxidative damage potentiation, atrophic gastritis development, autoimmune illness, delayed ulcer healing, and an increased risk of ulcer recurrence [86]. Smads are intermediary effector proteins that transfer signals from the cell surface straight into the nucleus, where they start transcription and play essential roles in TGF-beta’s biological activity. Enhancement of the TGF-β1/Smad pathway limited the inflammatory response associated with delayed wound healing [87]. The TGF-β1-Smad signaling pathway was also influenced by the interaction of other pathways, including the MAPK (Mitogen-Activated Protein Kinase), Wnt, and p53 circuits, and resulted in ulcer healing effects [88].

It was evident from the current findings that Phytol exerts its anti-inflammatory effect via direct inhibition of the inflammatory cytokines (like IL-6) and indirectly through enhancement of TGF-β1-mediated suppression of inflammation. These anti-inflammatory responses were potentiated by the nano-formulation of Phytol and combined with AMG (due to the involvement of NO).

Reactive oxygen species (ROS) and reactive nitrogen species (RNS) accumulate in the stomach tissue, causing oxidative stress and inflammation that damages the stomach mucosa. As a result, lipids and proteins are oxidized, and the permeability of the gut mucosa increases [89]. Ethanol consumption causes lipid peroxidation in the stomach tissue and increases the generation of reactive oxygen species (ROS), such as hydroxyl and superoxide anions. In parallel to the current results, previous research has shown that oxidative homeostasis, such as that mediated by glutathione (GSH), MDA, and superoxide dismutase (SOD), is involved in stomach ulcers caused by ethanol [90]. Pretreatment with Phytol (20 mg/kg) or nano-Phytol (20 mg/kg) showed noticeable protection against oxidative stress induced by ethanol ingestion. The antioxidant effect was more pronounced with nano-Phytol treatment and was comparable to the standard drug’s effect. This is consistent with the present findings that demonstrated the other mechanisms mediating the gastroprotective effect of the Phytol compound and their enhancement by nano-formulation. In parallel, earlier studies have described the role of the antioxidant effect of Phytol in ameliorating cognitive dysfunction [91] and streptozotocin-induced hyperglycemia as one of the constituents of the *Homalium Zeylanicum* plant [92]. It also presented a powerful *in vitro* antioxidant effect in thiobarbituric acid reactive species, hydroxyl radical scavenging activity, and scavenging activity of nitric oxide assays, and these effects were ascribed to the presence of the hydroxyl group. Phytol donates hydrogen atoms and converts free radicals into less reactive species [7].

In order to further explore the mechanistic pathways involved in Phytol’s gastroprotective effect, Western blotting analysis was performed. Ethanol cytotoxicity promotes the recruitment of inflammatory cytokines and leukocytes that release reactive oxygen species (ROS), all of which may hasten cell death [93]. NF-κB is crucial to understanding how these critical events relate to one another [94]. The inflammatory response and oxidative stress are regulated by the NF-κB, which is involved in dimerization, DNA binding, and interaction with inhibitory proteins [95]. Nuclear factor kappa-B (NF-κB) is a transcription factor that regulates the inflammatory response [93]. From the perspective of ethanol-induced gastric mucosal injury, NF-κB expression was increased, indicating its activation. Phytol and nano-Phytol treatments decreased NF-κβ expression, suggesting their anti-inflammatory effects. These findings indicate that Phytol may exert its gastroprotective effects by modulating the NF-κB signaling pathway, thus inhibiting inflammation, confirming the current biochemical findings.

Caspase-3 is a key mediator of apoptosis, and its activation can lead to cell death. Apoptosis is a key mechanism for maintaining cellular homeostasis [96]. The oxidative stress-induced apoptosis mediated by the anti-apoptotic and pro-apoptotic proteins serves a vital role in the degradation of gastric mucosal wall integrity after ethanol induction [97]. Inhibition of apoptosis via downregulation of apoptotic protein (caspase-3) alleviates gastric lesion recovery [97]. From the perspective of ethanol-induced gastric mucosal injury, caspase-3 expression was found to be upregulated, indicating increased apoptosis. However, treatment with Phytol downregulated caspase-3 expression, which suggests its potential anti-apoptotic effects. This finding highlights the role of Phytol in inhibiting apoptosis and promoting cell survival. It is worth noting that western blotting analysis showed the enhanced anti-inflammatory and anti-apoptotic effects of the formulation of nano-Phytol as reflected by increased inhibition of caspase-3 and NF-κβ.

Histopathological investigations in this study additionally confirmed the biochemical results. EtOH administration led to significant damage to the stomach lining, changed the configuration of gastric glands, and induced the infiltration of inflammatory cells. The results are consistent with previous published studies [98]. On the contrary, pretreatment of the rats with Phytol showed improvement in gastric lesions, and nano-Phytol restored the standard gastric mucosal architecture, especially the high dose of nano-Phytol (20 mg/kg) with no evidence of inflammation. This matches the above results, describing the ameliorative effect of nano-Phytol on gross ulcer number, severity, and biochemical parameters, and it explores its gastro-protective effect.

Meanwhile, the addition of L-NAME significantly exacerbated mucosal damage in rats with EtOH-induced ulcers and reduced the protective properties of Phytol in preventing stomach erosions and inflammation. This detrimental effect of L-NAME on gastric ulcers induced by other experimental models was described previously [67]. However, the combination with AMG promoted a Phytol-induced ulcer healing effect and nearly repaired the gastric mucosa. This proves that Phytol’s mode of action in rats involves the NO pathway. Collectively, both NO and TGF-β1 contribute to the cytoprotective effect of Phytol. The interaction of these two endogenous substances with their downstream signaling pathways seems essential for Phytol to successfully heal gastric ulcers by anti-oxidative, anti-inflammatory, angiogenic, and gastric pH, as well as improving blood flow mechanisms.

The CLSM findings deduced that the loading of Phytol in the alginate hydrogel beads caused significant effects on their physical, pharmacological, and biological stability characteristics. These results could be elucidated via (i) increasing the anti-ulcer activity, where the nano-Phytol showed better activity than Phytol itself, (ii) increasing the biological stability of nano-Phytol compared to Phytol itself, where the nano-Phytol penetration throughout the four layers of the stomach was stronger than Phytol itself. This penetration enhancement might be attributed to the small size of the as-synthesized nanoform (< 100 nm), which resulted in a much larger surface area. Moreover, the mucoadhesive characteristics of alginate beads, which extend the duration of medication in the stomach, could be beneficial for enhancing drug absorption and penetration to the deep layers compared to the raw Phytol.

## 5. Conclusions

Phytol administration protects the stomach against EtOH-induced gastric ulcers, as referred to by improved gross morphology, decreased ulcer number, severity, and better histopathological features. Nano-formulation of Phytol enhanced its gastro-protective effects in a dose-dependent manner, probably via cNOS activation and iNOS inhibition mechanisms. Suppression of inflammation (by inhibiting NF-κB & IL-6), oxidative stress, inhibition of apoptosis, and stimulation of TGF-β1 could be other mechanisms in the Phytol/nano-Phytol-induced healing effect of gastric ulcers. The development of Phytol nanoemulsions encapsulated in alginate hydrogel beads (Phytol@NE-HGBs), which showed higher penetration capacity into the different layers of the stomach and the notable anti-ulceration of Phytol and Phytol@NE-HGBs against the EtOH-induced rats *via* the NOS activation and NF-κB/IL-6/TGF-β modulation, was the work’s most significant achievement. The overall finding of the present work revealed that the development of Phytol nanoemulsions encapsulated in alginate hydrogel beads (Phytol@NE-HGBs) caused substantial enhancement of the anti-ulcer action and penetration through the stomach layers. These findings provide novel approaches to the global search for gastroprotective drugs with the lowest probable side effects. However, there were certain limitations, such as the requirement for further research on the anti-ulcer benefits of Phytol and Phytol@NE-HGBs against more ulcerative materials within their respective activity pathways.

## Supporting information

S1 Fig SEM of Phytol@NE-HGBs. (**A**) Cross-sectional morphology reveals the hydrogel bead structure. (**B**) Surface texture morphology, showing wrinkled yet defect-free bead surface topography.
(DOCX)

S2 Fig Raw image of Western blot analysis of Caspase 3. (DOCX)

S3 Fig Raw image of Western blot analysis of NF-κβ. (DOCX)

S4 Fig Raw image of Western blot analysis of β-actin. (DOCX)

## Abbreviations

AMG: Aminoguanidine
CCG: Carbon-Coated Copper Grids
CLSM: Confocal laser scanning microscopy
cNOS: Constitutive Nitric Oxide Synthase
DL: Drug Loading
DLS: Dynamic Light Scattering
EE: Entrapment Efficiency
EtOH: Ethanol
eNOS: Endothelial Nitric Oxide Synthase
FE-SEM: Field-Emission Scanning Electron Microscopy
GSH: Glutathione
H&E: Haematoxylin/Eosin
HGBs: Hydrogel Beads
HLB: Hydrophilic-Lipophilic Balance
IL-1β: Interleukin-1β
IL-6: Interleukin-6
IL-10: Interleukin-10
iNOS: Inducible Nitric Oxide Synthase
ip: Intraperitoneal
kV: Kilovolt
L-NAME: NG-nitro-L-Arginine Methyl Ester
M: Molar
MAPK: Mitogen-Activated Protein Kinase
MDA: Lipid peroxide
mV: Millivolt
NCs: Nanocarriers
NE: Nanoemulsion
NF-κB: Nuclear Factor Kappa-Light-Chain-Enhancer of Activated B Cells
NO: Nitric Oxide
nNOS: Neuronal Nitric Oxide Synthase
NOS: Nitric Oxide Synthase
NSAIDs: Non-Steroidal Anti-Inflammatory Drugs
p53: Tumor Suppressor Protein 53
PBS: Phosphate Buffer Saline
PDI: Polydispersity Index
Phytol@NE: Nanoemulsion Loaded with Phytol
Phytol@NE-HGBs: Phytol Nanoemulsions Encapsulated Alginate Hydrogel Beads
po: By mouth
PPAR: Peroxisome Proliferator-Activated Receptor
RCMB: Regional Center for Mycology and Biotechnology
RNSs: Reactive Nitrogen Species
ROS: Reactive Oxygen Species
rpm: Round Per Minute
SA-HGBs: Sodium Alginate Hydrogel Beads
SD: Standard Deviation
SEM: Standard Error of the Mean
SGF: Simulated Gastric Fluid
SIF: Simulated Intestinal Fluid
Smads: Intermediary Effector Proteins
TEM: Transmission Electron Microscopy
TGF-β: Transforming Growth Factor-β
TNF-α: Tumor Necrosis Factor-α
USP: United States Pharmacopeia
UV: Ultraviolet
VEGF: Vascular Endothelial Growth Factor
Wnt: Wnt protein
