# Supplementary material for: Phytol nanoemulsions encapsulated alginate hydrogel beads for the protection and management of alcohol-induced gastric ulcer via nitric oxide synthase and NF-κB/IL-6/TGF-β modulation
Source: PLoS One. 2025 Jul 11;20(7):e0327368. doi: 10.1371/journal.pone.0327368 (PMC12250206; doi:10.1371/journal.pone.0327368)
Supplement: S1 Fig — SEM of Phytol@NE-HGBs. (A) Cross-sectional morphology reveals the hydrogel bead structure. (B) Surface texture morphology, showing wrinkled yet defect-free bead surface topography. (DOCX) [file pone.0327368.s001.docx]

**
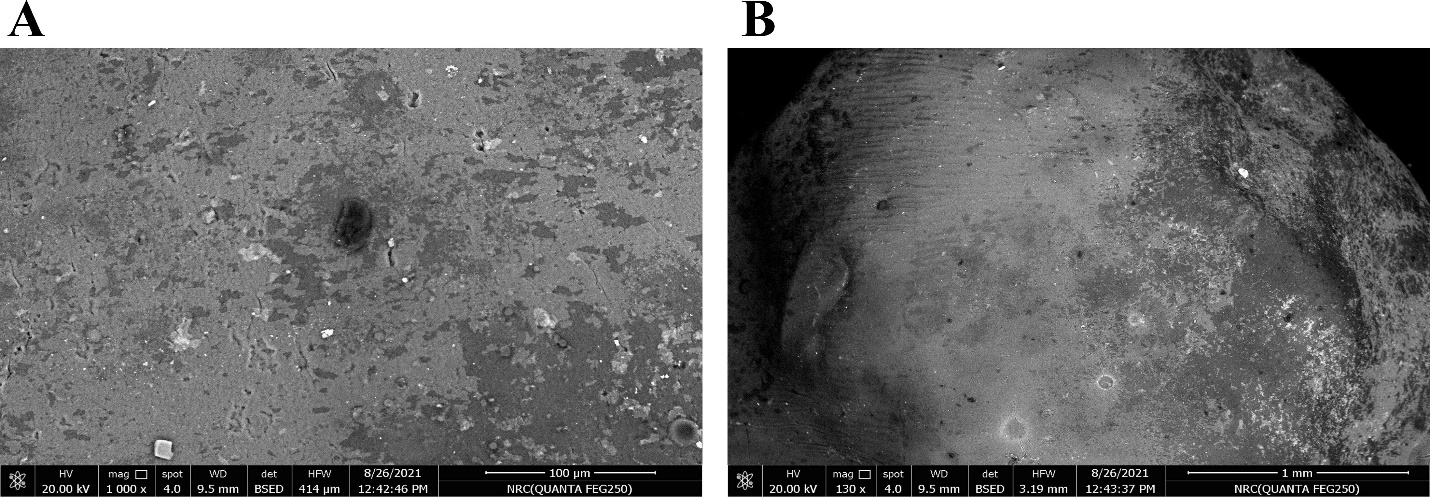
**

**Figure S1**: SEM of Phytol@NE-HGBs.

(**A**) Cross-sectional morphology reveals the hydrogel bead structure. (**B**) Surface texture morphology, showing wrinkled yet defect-free bead surface topography.
