# Supplementary figures and images for: Phytol nanoemulsions encapsulated alginate hydrogel beads for the protection and management of alcohol-induced gastric ulcer via nitric oxide synthase and NF-κB/IL-6/TGF-β modulation
Source: PLoS One. 2025 Jul 11;20(7):e0327368. doi: 10.1371/journal.pone.0327368 (PMC12250206; doi:10.1371/journal.pone.0327368)

**
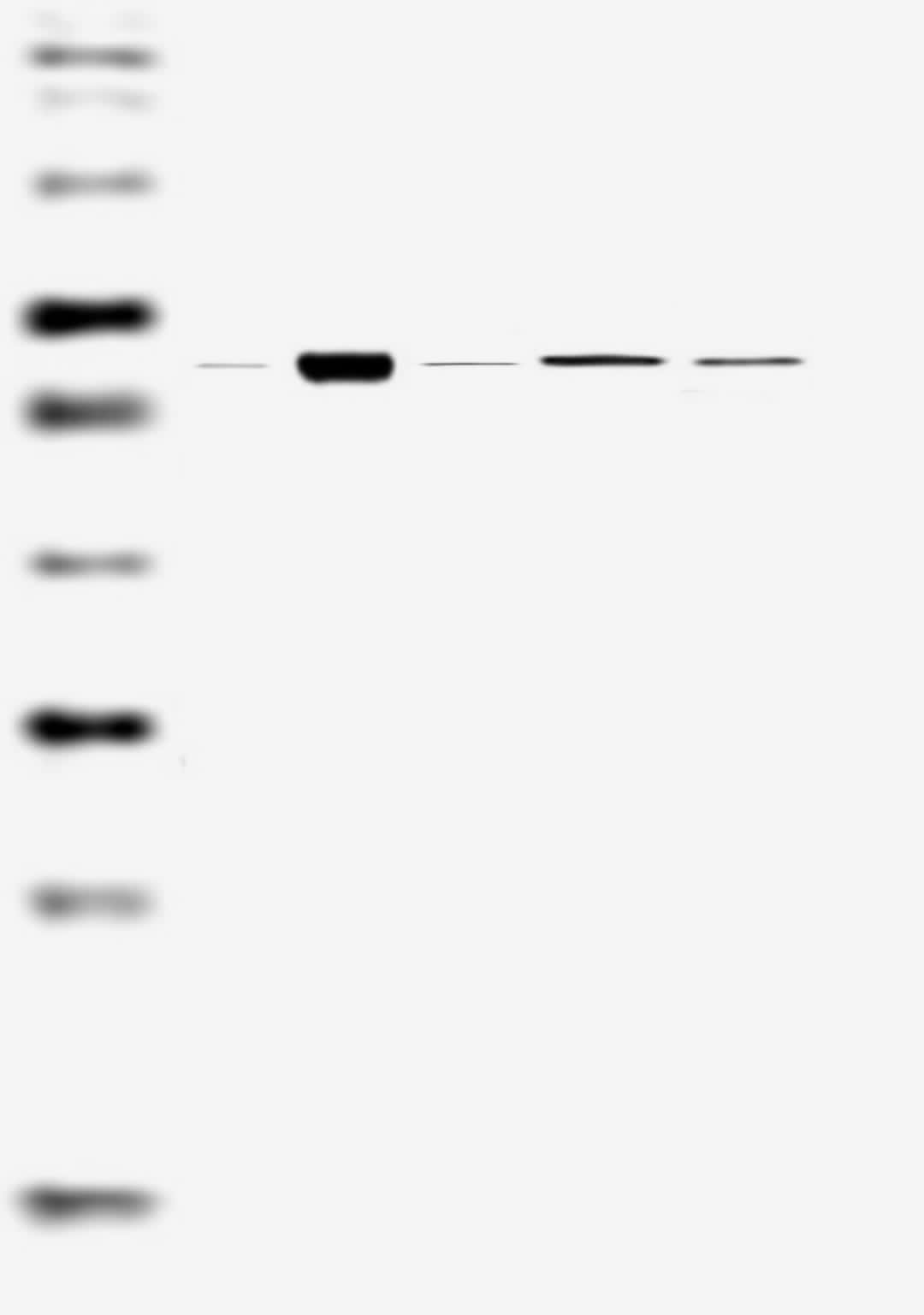
**

**Figure S2**: Raw image of Western blot analysis of Caspase 3.

Supplement: S2 Fig — (DOCX) [file pone.0327368.s002.docx]

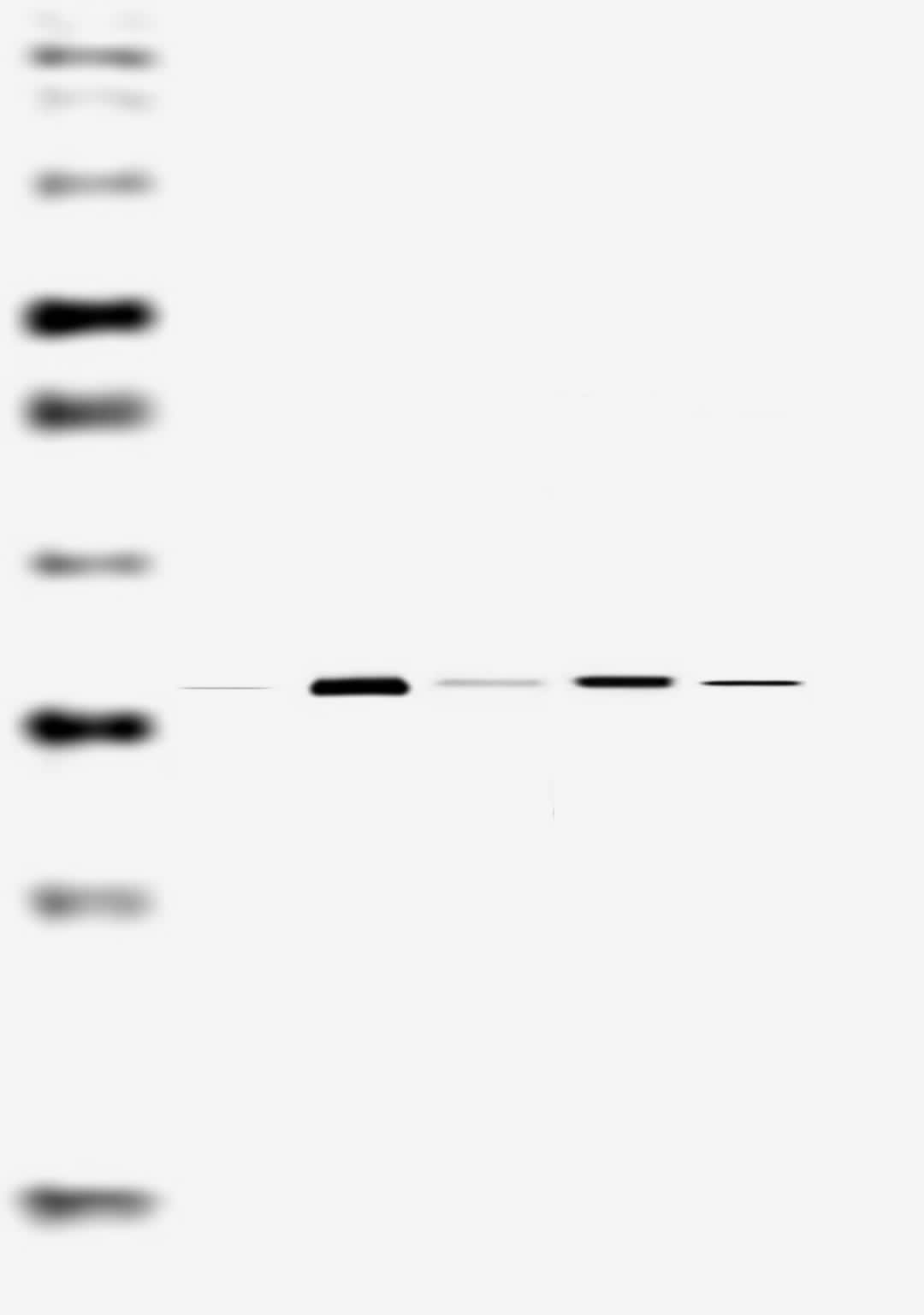


**Figure S3**: Raw image of Western blot analysis of NF-κβ.

Supplement: S3 Fig — (DOCX) [file pone.0327368.s003.docx]

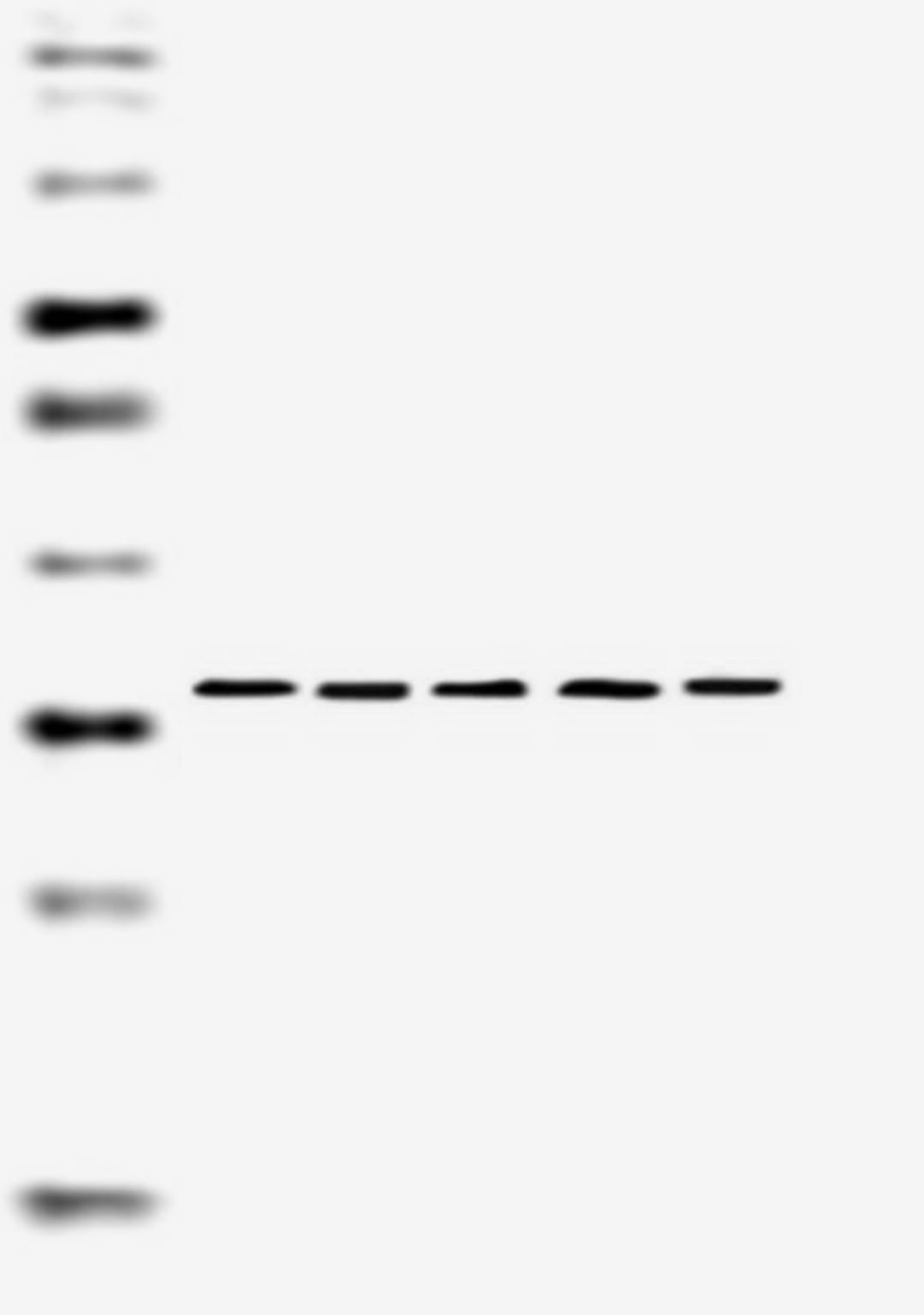


**Figure S4**: Raw image of Western blot analysis of β-actin.

Supplement: S4 Fig — (DOCX) [file pone.0327368.s004.docx]
